# Spatial proteomics and transcriptomics reveal early immune cell organization in pancreatic intraepithelial neoplasia

**DOI:** 10.1172/jci.insight.191595

**Published:** 2025-06-26

**Authors:** Melissa R. Lyman, Jacob T. Mitchell, Sidharth Raghavan, Luciane T. Kagohara, Amanda L. Huff, Saurav D. Haldar, Sarah M. Shin, Samantha Guinn, Benjamin Barrett, Gabriella Longway, Alexei Hernandez, Erin M. Coyne, Xuan Yuan, Lalitya Andaloori, Jiaying Lai, Yun Zhou Liu, Rachel Karchin, Anuj Gupta, Ashley L. Kiemen, André Forjaz, Denis Wirtz, Pei-Hsun Wu, Atul Deshpande, Jae W. Lee, Todd D. Armstrong, Nilofer S. Azad, Jacquelyn W. Zimmerman, Laura D. Wood, Robert A. Anders, Elizabeth D. Thompson, Elizabeth M. Jaffee, Elana J. Fertig, Won Jin Ho, Neeha Zaidi

**Affiliations:** 1 Department of Oncology, Sidney Kimmel Comprehensive Cancer Center,; 2 Convergence Institute,; 3 Bloomberg Kimmel Immunology Institute,; 4 Department of Genetic Medicine,; 5 Department of Biomedical Engineering,; 6 Computational Medicine,; 7 Department of Pathology,; 8 Department of Chemical and Biomolecular Engineering,; 9 Department of Materials Science and Engineering, and; 10 Department of Applied Mathematics and Statistics, Whiting School of Engineering, Johns Hopkins University, Baltimore, Maryland, USA.; 11 Institute for Genome Sciences, Department of Medicine, and Greenbaum Comprehensive Cancer Center, University of Maryland School of Medicine, Baltimore, Maryland, USA.

**Keywords:** Immunology, Inflammation, Oncology, Adaptive immunity, Cancer immunotherapy, Gastric cancer

## Abstract

Pancreatic ductal adenocarcinoma (PDAC) has a poor survival rate due to late detection. PDAC arises from precursor microscopic lesions, termed pancreatic intraepithelial neoplasia (PanIN), that develop at least a decade before overt disease; this provides an opportunity to intercept PanIN-to-PDAC progression. However, immune interception strategies require full understanding of PanIN and PDAC cellular architecture. Surgical specimens containing PanIN and PDAC lesions from a unique cohort of 5 treatment-naive patients with PDAC were surveyed using spatial omics (proteomic and transcriptomic). Findings were corroborated by spatial proteomics of PanIN and PDAC from tamoxifen-inducible KPC mice. We uncovered the organization of lymphoid cells into tertiary lymphoid structures (TLSs) adjacent to PanIN lesions. These TLSs lacked CD21^+^CD23^+^ B cells compared with more mature TLSs near the PDAC border. PanINs harbored mostly CD4^+^ T cells, with fewer Tregs and exhausted T cells than PDAC. Peritumoral space was enriched with naive CD4^+^ and central memory T cells. These observations highlight the opportunity to modulate the immune microenvironment in PanINs before immune exclusion and immunosuppression emerge during progression into PDAC.

## Abstract 2

Deep profiling of precursor lesions to pancreatic cancer reveal processes of a changing immune response that present targets for therapeutic interception.

## Introduction

Pancreatic ductal adenocarcinoma (PDAC) carries a dismal 5-year survival rate of 12%, due to late-stage diagnosis and resistance to standard therapies and immune checkpoint inhibitors (ICIs) (1–3). PDAC is complex, with few infiltrating effector T cells, and an abundance of immunosuppressive regulatory T cells (Tregs), myeloid-derived suppressor cells, tumor-associated macrophages, and cancer-associated fibroblasts, establishing an immunologically “cold” tumor microenvironment (TME) (4–9).

Precancer lesions — pancreatic intraepithelial neoplasia (PanINs) — are initiated from driver mutations in *KRAS* (10). It takes years to decades for invasive PDAC to develop after the first mutation, providing a protracted window to intercept precancer progression (10). Previous studies provide evidence for decreased immunosuppressive signaling and increased T cell trafficking in mouse and human PanINs (11, 12). We reported, for the first time to our knowledge, that a *Listeria*-based mKRAS^G12D^ vaccine with Treg-depleting agents triggered mKRAS-specific T cell responses and slowed early- but not late-stage PanIN-to-PDAC progression in mice (13). While these studies highlight the potential therapeutic benefit of early vaccination against driver mutations in PanINs to prevent or delay PDAC progression, little is known about the human precancer microenvironment (PCE) that accompanies PDAC.

Procurement of large sections of human pancreatic tissue containing PanIN independent of PDAC has proven infeasible, as PanINs are not detectable by imaging to justify tissue resection. Therefore, we utilized resected PDAC tissue to study distinct PanIN regions that oftentimes exist within the resected tissue specimen. Healthy human pancreases may harbor genetically diverse PanINs (14), most of which likely do not progress to PDACs. Since PanINs are microscopic lesions, spatial proteomics and transcriptomics are required to fully resolve the PCE. Spatial profiling offers a unique opportunity to quantitate cell-cell organization and interaction within the PCE, while maintaining the cellular architecture of immune cells. The latter analysis is relevant to tertiary lymphoid structures (TLSs), which are organized aggregates of lymphoid cells typically in peritumoral regions of untreated PDAC (15). Peritumoral TLSs in untreated PDACs appear inactive, with few effector T cells. However, TLSs have been induced to traffic into tumors following treatment with an allogenic whole-cell-based GM-CSF vaccine, GVAX (16). These treatment-induced TLSs associated with a survival benefit appear similar to those found naturally in ICI-responsive tumors (15, 17, 18). However, little is known about TLSs that develop in precancers.

Here, we utilized spatial proteomics and transcriptomics to characterize the PCE. Our analysis leverages a unique cohort of untreated resected PDAC specimens from patients with distinct regions of normal tissue, chronic pancreatitis (CP), and PanINs within the same tissue section. We report, for the first time to our knowledge, the recruitment and organization of lymphoid cells into immature TLSs during early PanIN development. The spatially resolved data also show structurally and functionally mature and immature TLSs in the peritumoral regions where immune cells are largely restricted to the tumor border. Furthermore, we find that immune populations excluded from the tumor are denser and enriched with more naive and exhausted CD4^+^ T cells and antigen-experienced CD8^+^ T cells than within the tumor boundary.

While these spatial analyses can define immune subpopulations and uncover their function at the time of resection, it is not possible to evaluate PanIN-to-PDAC progression in human biospecimens, which would require serial biopsies of the same lesions over years. To assess whether immune cell states identified in the human PCE are present before progression to PDAC, we used the tamoxifen-inducible KPC (tiKPC) mouse (19), where PanINs progress to PDAC in a predictable timeframe. We find that features of T cell accumulation in human specimens are recapitulated in the tiKPC mice. We also show that organized lymphoid cell recruitment and precursor lymphoid aggregates appear in PanINs in mice. Thus, these studies support a pattern of lymphoid aggregate development and maturation in association with PanIN-to-PDAC development and suggest that the earliest aggregates contain a proimmune population that is targetable by immunotherapeutics to intercept tumorigenesis.

## Results

### Spatial multiomics profiling of lymphoid cells demonstrates CD4^+^ T cells recruited to clonally distinct PanIN regions relative to PDAC in treatment-naive patients.

**Figure 1:**
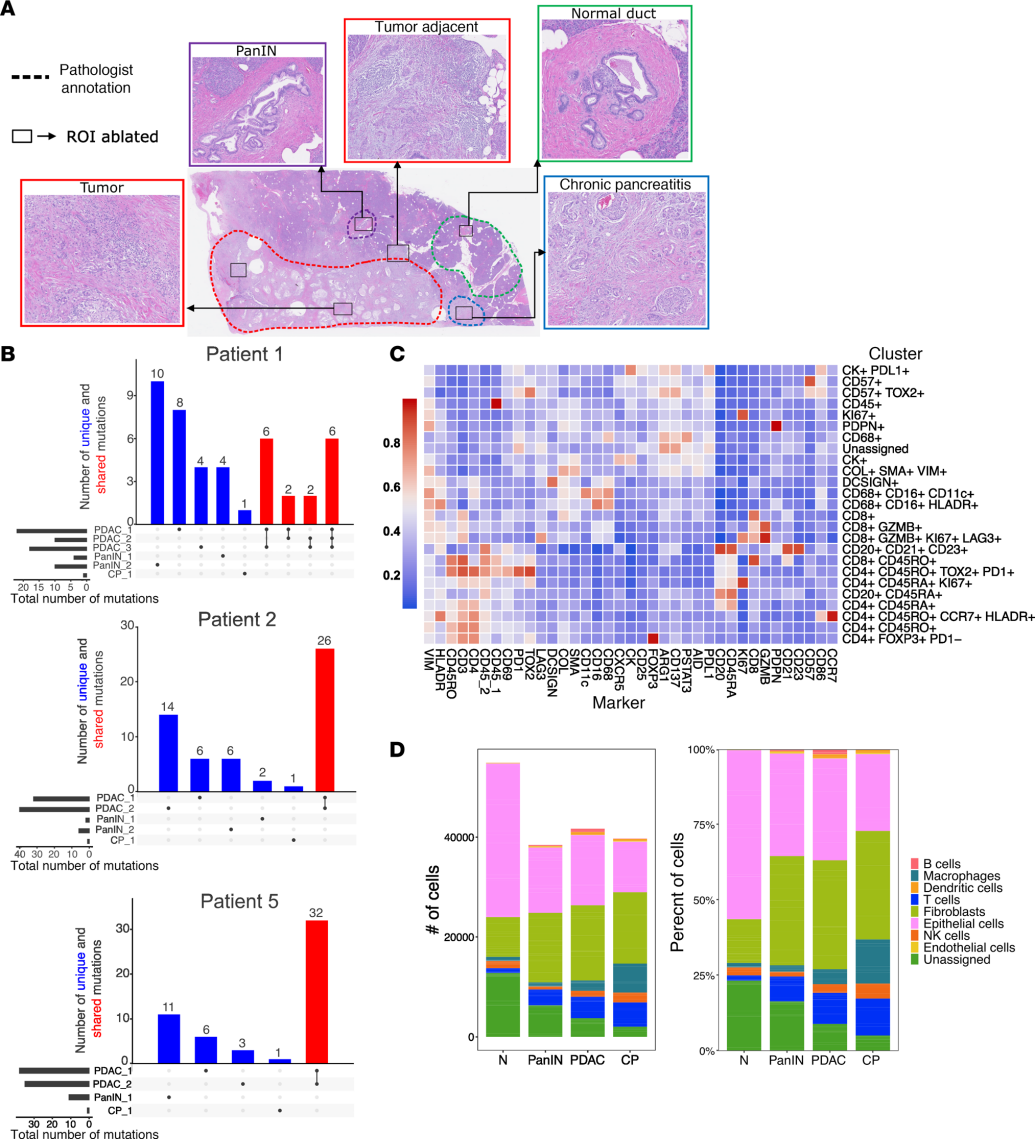
Immune-rich regions were identified and segmented for IMC analysis. (**A**) Visualization of the 4 different regions (normal, low-grade PanIN, PDAC, chronic pancreatitis) annotated by an expert pathologist. (**B**) Bar plots of the number of unique and shared mutations between PanIN lesions and PDAC from patients 1, 2, and 5. (**C**) Heatmap showing the relative expression of each marker in the IMC panel used to identify each cell type. (**D**) Total number of cells analyzed by region (left) with visualization of the percentage distribution of cells per region (right) (total area of tissue and number of samples used in the subsequent analysis is summarized in Supplemental Table 2).

To determine immune profiles near PanINs unaltered by prior therapies, we analyzed resected pancreases from 5 treatment-naive patients for which PanINs and chronic inflammation resided in areas distinct from tumor cells (Figure 1A). To evaluate the genetic relationship between PanIN and the PDAC loci on shared tissue sections, we used laser capture microdissection to extract DNA from normal, PanIN, PDAC, and CP regions. Whole-exome sequencing of 15 regions from 3 samples showed that PanINs were clonally distinct from associated tumors, as in Braxton et al. (14) (Figure 1B). Mutations were shared among different PDAC regions within each patient, but regions of PanIN had independent mutations not shared with other regions of PanIN or PDAC. PanINs in 3 of 4 patients exhibited oncogenic *KRAS* mutations that differed from PDAC found in the same resection (Supplemental Table 1; supplemental material available online with this article; https://doi.org/10.1172/jci.insight.191595DS1), establishing intrapatient precancer heterogeneity.

We used imaging mass cytometry (IMC) to label human tissue sections with a panel of 38 antibodies (Supplemental Table 2). To identify regions of interest (ROIs), an expert pathologist selected patient tissue blocks that were cut and annotated (Figure 1A and Supplemental Figure 1A). We analyzed 4 specific ROIs that included PanIN, PDAC, CP, and normal tissue (Figure 1A and Supplemental Figure 1B). A caveat to this analysis is the choice of the relatively uncommon T cell–rich areas within tumor regions. Relative immune-rich regions within the boundaries of tumors were chosen when possible and identified using immunohistochemistry (IHC) for CD3^+^ T cells (Supplemental Figure 2). Upon image acquisition, we confirmed the morphologies of pancreatic ductal cells near each immune-rich region by IMC to classify normal, PanIN, PDAC, and CP regions (Supplemental Figure 3).

Clustering analysis of the IMC markers identified epithelial cells, fibroblasts, myeloid, and lymphoid populations as the dominant cell types (Figure 1, C and D). All structural components of pancreatic tissue and immune cells were classified based on the relative expression profile of each IMC marker across the clusters (Figure 1C). While our focus was on the lymphoid cells, we also identified macrophages (CD68^+^), dendritic cells (DCSIGN^+^), epithelial cells (CK^+^), fibroblasts (COL^+^SMA^+^VIM^+^), endothelial cells (PDPN^+^), and NK cells (CD57^+^) (Figure 1D). The total cell number and composition showed that normal regions contained the highest number of cells (Figure 1D), not unexpectedly, as the total area analyzed from normal tissue was greater than other regions (Supplemental Table 4). Many of these cells make up acinar tissue or islets accounting for an “unassigned” cluster of cells in normal regions, which was confirmed visually by mapping their (*x*, *y*) coordinates and directly comparing to both the reference hematoxylin and eosin (H&E) images and MCD files. Patient-specific contribution to these cell types was variable, but consistent with the total area of tissue analyzed for each sample (Supplemental Figure 4A and Supplemental Tables 4 and 5). Principal component analysis showed no patient-specific outliers (Supplemental Figure 4B).

**Figure 2:**
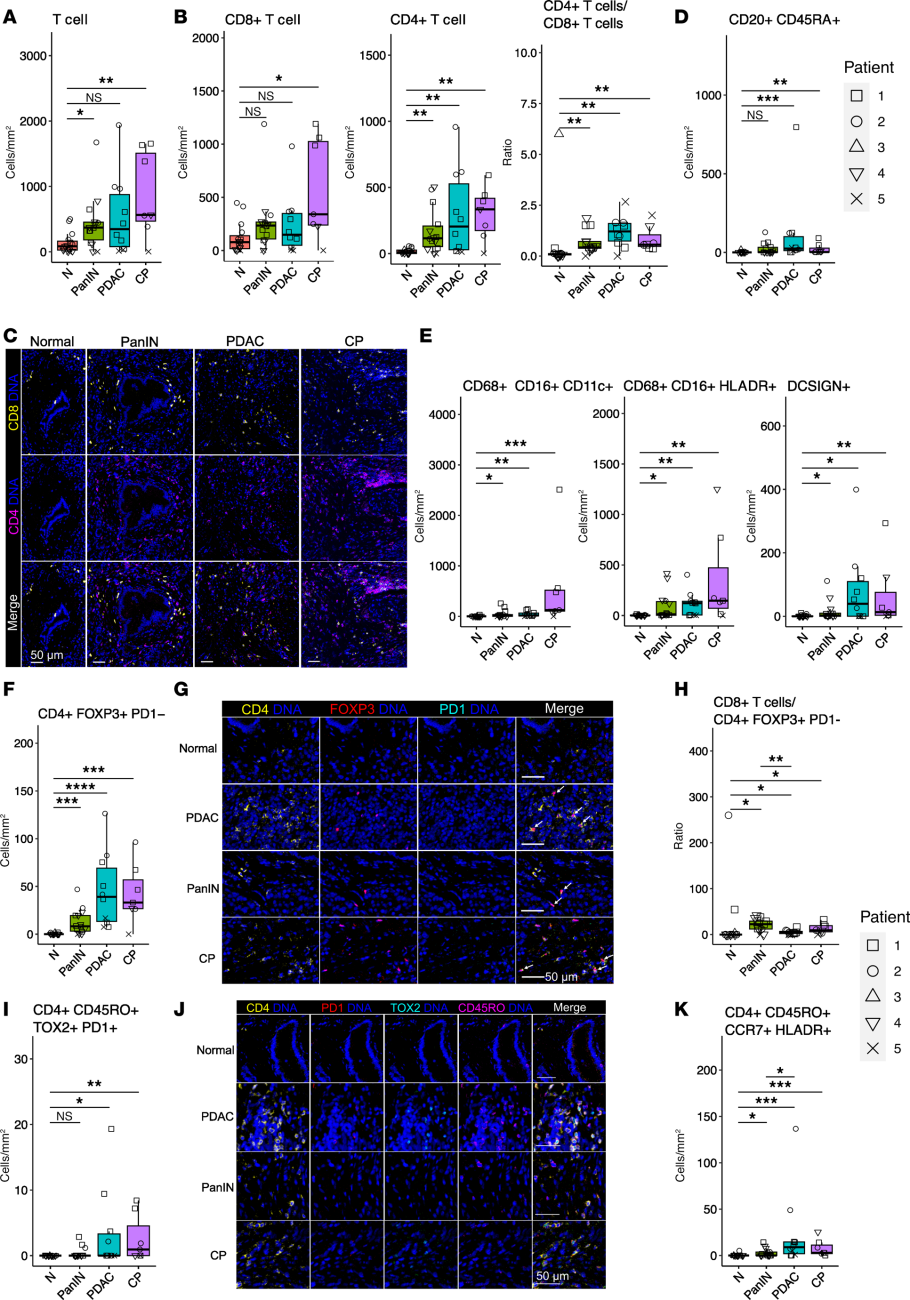
PanINs exhibit reduced exhausted and Treg populations relative to regions of PDAC. (**A**) Box plot of CD3^+^ T cell density. (**B**) Box plots of the density of CD4^+^ and CD8^+^ T cells, and the ratio of CD4^+^ T cells over CD8^+^ T cells. (**C**) Representative images of the CD4^+^ and CD8^+^ populations in normal, PanIN, PDAC, and CP regions. (**D**) Box plot of the CD20^+^CD45RA^+^ B cell density. (**E**) Box plots of the macrophage and dendritic cell densities (CD68^+^CD16^+^CD11c^+^, CD68^+^CD16^+^HLADR^+^, DCSIGN^+^). (**F**) Box plot of Treg (CD4^+^FOXP^+^PD1^+^) density. (**G**) Representative images of the Treg population in each region: normal, PanIN, PDAC, and CP. (**H**) Box plot of the ratio of CD8^+^ T cells over the Treg population. (**I**) Box plot of the density of CD4^+^CD45RO^+^TOX2^+^PD1^+^ or exhausted CD4^+^ T cells. (**J**) Representative images of the exhausted T cell population in each region: normal, PanIN, PDAC, and CP. (**K**) Box plot of the central memory CD4^+^ T cell (CD4^+^CD45RO^+^CCR7^+^HLADR^+^) density. White arrows on merged representative image panels indicate the cell type of interest. Densities were calculated using the number cells per mm^2^ of each ROI. Scale bars: 50 μm. Significance was determined using Wilcoxon’s rank-sum test. *P ≤ 0.05; **P ≤ 0.01; ***P ≤ 0.001; ****P ≤ 0.0001. NS, *P* > 0.05.

Total T cell (CD3^+^) density in PanIN and CP was significantly greater than regions of normal pancreas, whereas there was a trend in PDAC (Figure 2A). Compared with patient-matched normal tissue, immune infiltration in PanIN, PDACs, and CP was predominantly CD4^+^ T cells (Figure 2, B and C), consistent with studies of PanINs that were not compared with associated PDAC (12). While CD4^+^ T cells accounted for most of this increase, CD8^+^ T cells in CP were also elevated relative to normal tissue (Figure 2B). B cell (CD20^+^CD45RA^+^) density was also elevated in PDAC and CP, with a variable trend in PanINs (Figure 2D). Macrophage and dendritic cell (CD68^+^CD16^+^CD11c^+^, CD68^+^CD16^+^HLADR^+^, DCSIGN^+^) densities were increased in PanIN, PDAC, and CP regions compared with normal tissue across all matched patient samples (Figure 2E). Collectively, the findings demonstrate predominantly CD4^+^ T cell infiltration in low-grade PanINs.

### PanINs harbor fewer Tregs compared with PDACs.

IMC offers both spatial resolution and in-depth phenotypic delineation of T cells in PanINs and PDACs. We found that Tregs (CD4^+^FOXP3^+^PD1^–^) were significantly greater in all 3 pathologies compared with normal ductal tissue (Figure 2, F and G). As expected, Treg density was higher in PDACs than in PanINs, suggesting an early ingress of Tregs into the PanIN PCE. Despite the presence of Tregs in low-grade PanINs, the ratio of CD8^+^ T cells to Tregs was higher in PanINs than in PDACs (Figure 2H). The relatively few immunosuppressive cells in PanINs indicates a more amenable environment for inducing cytotoxic CD8^+^ T cells and mitigating the early immunosuppressive effects of Tregs. Moreover, there was a minimal exhausted CD4^+^ T cell population (CD4^+^CD45RO^+^TOX2^+^PD1^+^) in PanIN compared with PDAC or CP (Figure 2, I and J), expectedly, given that T cell exhaustion occurs from continued antigen or inflammatory signal exposure. Compared with normal tissue, the increase in the central memory T cell population (CD4^+^CD45RO^+^CCR7^+^HLADR^+^) was marginal in PanIN regions but increased in PDAC and CP (Figure 2K). Together, these data suggest that PanIN lesions harbor fewer immunosuppressive signals and higher quality T cells compared with PDAC.

We calculated the shortest distance between each cell in each ROI and depicted the relative number and distance in an interaction network (Supplemental Figure 5). Most immune cells in normal, PanIN, and CP were positioned closely to stromal fibroblasts (COL^+^SMA^+^VIM^+^). In contrast, PDAC intratumoral regions exhibited lymphoid cells in close proximity to one another, indicating more frequent interactions. Interestingly, the Tregs (CD3^+^CD4^+^FOXP3^+^PD1^–^) in PDAC regions were much more spatially integrated with immune cells, particularly closer to the cytotoxic T cells (CD3^+^CD8^+^GZMB^+^), compared with PanIN and CP. These data not only suggest closer communication, and possibly, more frequent interactions between Tregs and cytotoxic CD8^+^ T cells, but also further support the role of Tregs in dampening the antitumor immunity exerted by the rare, immune-rich regions spatially adjacent to PDAC.

Immune cells were largely restricted to the tumor front within 250 μm of the leading invasive edge (Supplemental Figure 6A). Notably, there was a higher density of naive and exhausted CD4^+^ T cells, and antigen-experienced CD45RO^+^CD8^+^ T cells. By measuring distances between each cell in the immune-rich border, we generated an interaction network (Supplemental Figure 6B). These lymphoid cells were again positioned closely together, but with greater distances from Tregs (CD3^+^CD4^+^FOXP3^+^PD1^–^) compared with interactions depicted in the intratumoral lymphoid population.

### Spatial proteomics of lymphoid cell populations uncovers organized and distinct TLSs unique to PanIN versus PDAC.

**Figure 3:**
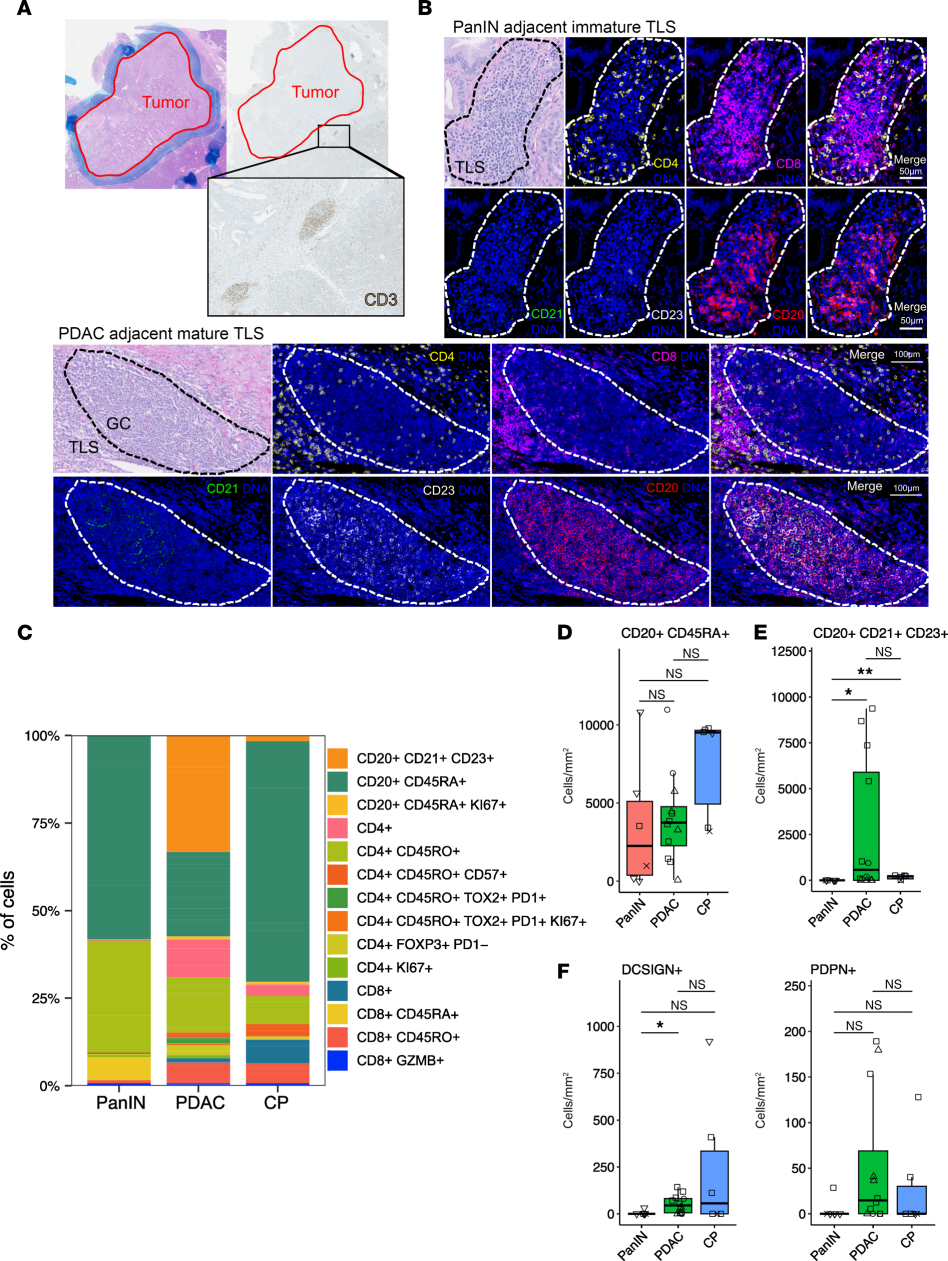
Tertiary lymphoid structures (TLSs) associated with PanINs maintain an immature phenotype compared with a mature phenotype in peritumoral TLSs. (**A**) H&E staining of PDAC region annotated in blue marker by an expert pathologist, with the outline of the tumor region overlaid on an IHC stain for CD3^+^ (brown) of a serial section of the same tissue, demonstrating the localization of the lymphoid aggregates in the peritumoral region. (**B**) Representative H&E staining of a TLS found within 250 μm of the invasive edge of the tumor and one within 250 μm of the edge of a PanIN and corresponding IMC staining of CD20^+^, CD21^+^, CD23^+^, CD4^+^, and CD8^+^ cells, indicating a mature TLS adjacent to PDAC and an immature TLS adjacent to PanIN. In PanIN-adjacent TLS images, the entire TLS is circled with a dashed white line, while in the PDAC-adjacent TLS images, the germinal center is circled with a dashed white line. Scale bars: 50 μm (top 2 rows) and 100 μm (bottom 2 rows). (**C**) Percentage distribution of lymphoid cell types according to the region of each TLS included in the analysis. (**D**) Box plot of the density of CD20^+^CD45RA^+^ B cells in PanIN-, PDAC-, and CP-adjacent TLSs. (**E**) Box plot of the density of germinal center–associated B cells (CD20^+^CD21^+^CD23^+^). (**F**) Box plots of the density of dendritic cells (DCSIGN^+^) and high endothelial venules (PDPN^+^) in each region-specific TLS. Densities were calculated using the number of cells per mm^2^ of each ROI. Significance was determined using Wilcoxon’s rank-sum test. *P ≤ 0.05; **P ≤ 0.01. NS, *P* > 0.05.

In many immune-rich regions at the tumor front, we noted aggregated clusters of CD3^+^ cells (Figure 3A). IMC showed that these aggregates contained cell types and a structural organization consistent with mature TLSs, as defined by a distinct zone of B cells (CD20^+^) with markers of follicular cells (CD21^+^CD23^+^) and surrounded by a dense zone of CD4^+^ and CD8^+^ T cells (20) (Figure 3B). We also found immature TLSs adjacent to PanIN (within 250 μm of the epithelial cell edge), defined as dense but disorganized aggregates of B and T cells without follicular dendritic cells or cell-type-specific zones. Both mature and immature TLSs were also found in CP regions (Supplemental Table 6).

We studied the diversity and organizational differences across these TLSs using histoCAT (21), an interactive platform for visualizing multiplexed IMC images. Individual lymphoid aggregates were separated into individual samples from the rest of the cells captured in a given region, allowing for the identification of differences specific to TLSs, but independent from the surrounding immune infiltrate. Profiling 27 TLSs by IMC (Supplemental Table 5) revealed that PDAC- and CP-adjacent TLSs comprised a more diverse subset of B and T cells compared with PanIN-adjacent T cells (Figure 3C). Compared with PDAC, TLSs in PanIN regions were associated with fewer total cells (Supplemental Table 7). Supplemental Table 8 details the number of TLSs per patient and per region. We excluded TLSs not within 250 μm of (a) the edge of epithelial cells comprising PanINs, (b) the CP region, or (c) invasive tumor border as defined and measured by a pathologist (Supplemental Figure 7).

For the most predominant B cell subpopulation (CD20^+^CD45RA^+^), density was not significantly different across PanIN-, PDAC-, or CP-associated TLSs (Figure 3D). There were significant differences in the density of germinal center–associated B cells (CD20^+^CD21^+^CD23^+^), with increases specific to PDAC-associated TLSs (Figure 3E). This population was absent in PanIN-associated TLSs and minimal in CP-associated TLSs, indicative of maturity specific to some PDAC- and CP-associated TLSs (22, 23). We also found other indications of mature TLSs associated with PDAC and CP regions based on the densities of dendritic cells (DCSIGN^+^) and high endothelial venules (PDPN^+^) in TLSs associated with PDAC and CP (Figure 3F).

### PDAC-adjacent TLSs have distinct T cell subtypes compared with TLSs associated with PanIN.

**Figure 4:**
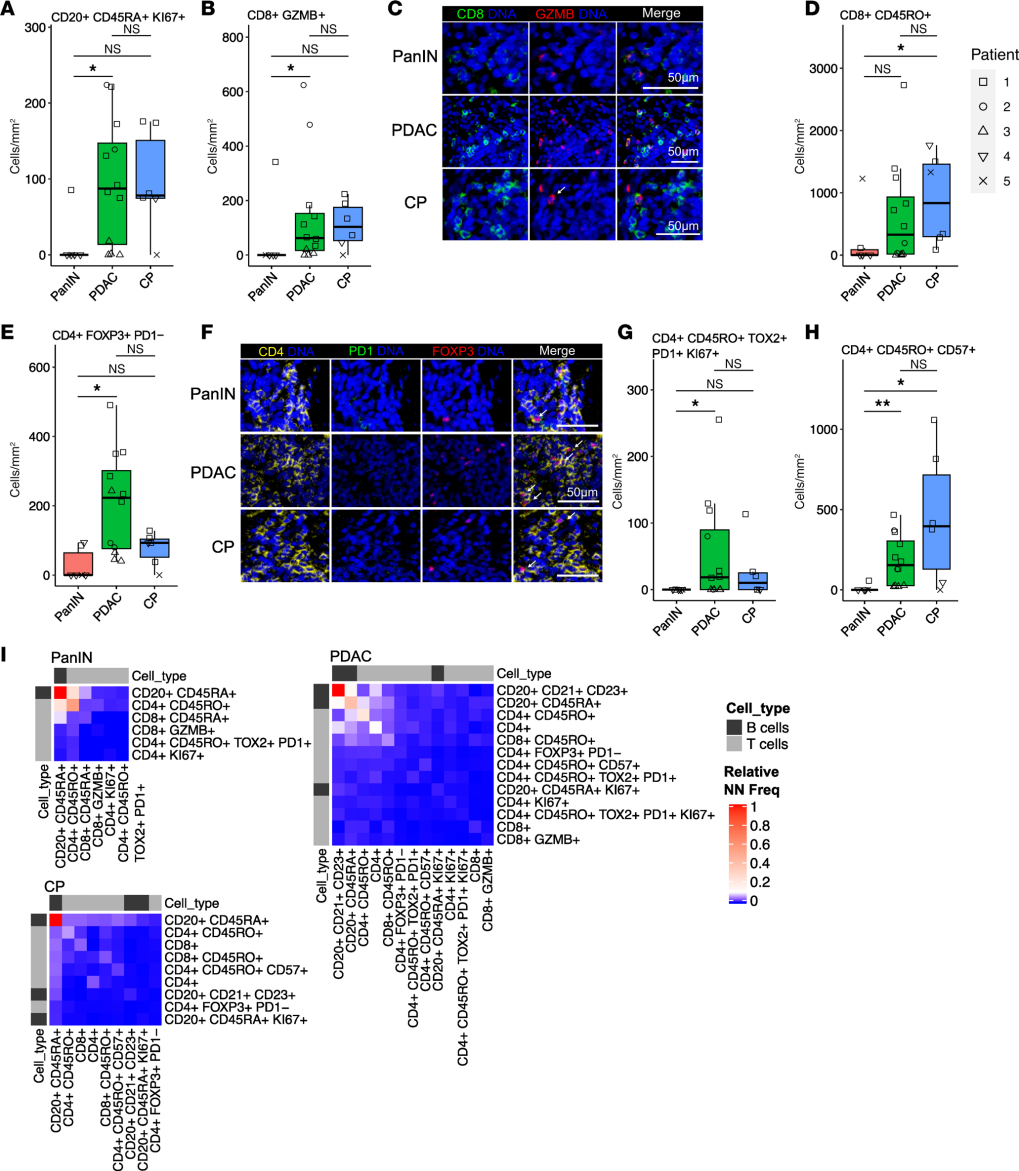
PDAC-adjacent TLSs have distinct proteomic signatures compared with TLSs adjacent to PanINs. (**A**) Box plot of proliferative B cell (CD20^+^CD45RA^+^KI67^+^) densities. (**B**) Box plot of the density of cytotoxic CD8^+^ T cells (CD8^+^GZMB^+^). (**C**) Representative images of the cytotoxic T cell population in PanIN-, PDAC-, and CP-adjacent TLSs. (**D**) Box plot of the densities of antigen-experienced CD8^+^ T cells (CD8^+^CD45RO^+^). (**E**) Box plot of Treg (CD4^+^FOXP3^+^PD1^–^) density. (**F**) Representative images of the Treg population in PanIN-, PDAC-, and CP-adjacent TLSs. (**G**) Box plot of the density of exhausted T cells (CD4^+^CD45RO^+^TOX2^+^PD1^+^KI67^+^). (**H**) Box plot of the density of senescent CD4^+^ T cells (CD4^+^CD45RO^+^CD57^+^). (**I**) Nearest neighbor analyses of the top 2 nearest neighbors of each lymphoid subtype. Red denotes a greater frequency of a neighboring cell type, whereas blue denotes a less frequent neighboring cell types. White arrows on merged representative image panels indicate the cell type of interest. Densities were calculated using the number of cells per mm^2^ of each ROI. Scale bars: 50 μm. Significance was determined using Wilcoxon’s rank-sum test. *P ≤ 0.05; **P ≤ 0.01. NS, *P* > 0.05.

PDAC-adjacent TLSs displayed higher expression of activation and effector molecules on T and B cells compared with PanIN-adjacent TLSs. There was a significantly higher density of proliferating B cells (CD20^+^CD45RA^+^KI67^+^) in PDAC TLSs compared with PanIN- and CP-adjacent TLSs (Figure 4A). Cytotoxic T cell (CD3^+^CD8^+^GZMB^+^) density was also higher in peritumoral TLSs, while the CD3^+^CD8^+^CD45RO^+^ T cells were trending higher compared with PanIN-associated TLSs (Figure 4, B–D). While this indicates more activated T cells, there were also more Tregs (CD3^+^CD4^+^FOXP3^+^PD1^–^) and exhausted T cells (CD4^+^CD45RO^+^TOX2^+^PD1^+^KI67^+^) populating PDAC-adjacent TLSs (Figure 4, E–G). Lastly, PDAC- and CP-associated TLSs had a greater memory CD4^+^ T cell (CD4^+^CD45RO^+^CD57^+^) density (Figure 4H). Collectively, the data show that organization of recruited lymphoid cells begins at early stages of carcinogenesis, and that these cells appear antigen-experienced and exhausted, with localization just outside the tumor border and proximal to Tregs. The data also demonstrate a less immunosuppressive and exhausted T cell phenotype in TLSs associated with precancers.

The spatial organization of subtypes provides further evidence for the structural and phenotypic maturity of the PDAC and CP TLSs compared with PanIN-associated TLSs. By identifying the top 2 nearest neighbors of each lymphoid cell, we determined which cell types are in most frequent contact (and likely interacting). We observed that germinal center B cells are frequently in contact with other B cells and T cells in PDAC-associated TLSs (Figure 4I). Whereas TLSs near PanIN and CP had B cells and memory helper T cells (CD4^+^CD45RO^+^) in close contact with naive-appearing cytotoxic T cells (CD8^+^CD45RA^+^), PDAC-adjacent TLSs displayed B cells and memory helper T cells in contact more frequently with more differentiated cytotoxic T cells (CD8^+^CD45RO^+^) and Tregs (CD4^+^FOXP3^+^). Neighbor analyses further confirmed (a) the existence of true germinal centers unique to the PDAC-adjacent TLSs that are notably absent in any PanIN-adjacent TLS, and (b) that PanIN-adjacent TLSs reflect earlier steps in the formation of immune responses with less immunosuppression.

### PanIN formation in tiKPC mice recapitulate accumulation of Tregs and lymphoid aggregates as a dynamic change associated with PDAC carcinogenesis.

Although profiling the immune microenvironment of human PanINs is critical to understanding PDAC progression, these lesions are detected only by PDAC resection. While we hypothesize that spatially distinct lesions are independent, allowing spatial distance and genetic differences to serve as surrogate for the evolution of the disease, we are limited in our ability to directly infer the timing of immunological changes during carcinogenesis. To overcome this limitation in temporal sampling, we used a tamoxifen-inducible genetically engineered mouse model — the *Kras*^LSL-G12D^;*p53*^LoxP^;*Pdx1*-*Cre*ER (tiKPC) mouse (19) — to generate and harvest PanIN lesions in vivo before development into PDAC.

Tumor development was induced by daily intraperitoneal injections of tamoxifen over 5 days, followed by sacrifice at 10–16 weeks after induction (Supplemental Figure 8A). Whole pancreases from 11 mice were sectioned and representative regions of normal duct, PanIN, and PDAC identified by a pathologist. We noted increased T cell infiltration in PanIN and PDAC relative to normal ducts, as well as lymphoid aggregates proximal to PanIN and PDAC (Supplemental Figure 8C). The latter lacked the organized germinal centers characteristic of mature human TLSs, possibly due to the short antigen exposure.

Sections of mouse pancreas were analyzed using IMC (Supplemental Table 3). Protein expression in segmented cells was used to annotate cells by hierarchical clustering (Supplemental Figure 8B). We inferred the following T cell phenotypes: CD4^+^ T helper, CD4^+^ Treg, and CD8^+^ T cells. ROIs were annotated by segmentation with histoCAT (21) as normal (*n* = 12), PanIN (*n* = 8), tumor edge (*n* = 18), tumor core (*n* = 16), and lymphoid aggregate (PanIN-adjacent, *n* = 4, PDAC-adjacent, *n* = 5) (Supplemental Figure 8C). T cell densities, including all 3 observed phenotypes, were compared between regions.

PanINs were enriched for T cells compared with normal tissue, as were the tumor edges, upon excluding lymphoid aggregates (Supplemental Figure 8D). Tumor cores displayed lower T cell densities compared with PanIN. Normal ductal tissues had lower enrichment of CD8^+^ T cells compared with PanIN, tumor edge, or tumor core. No significant difference in CD8^+^ T cell density was noted across the tumor regions (Supplemental Figure 8E). Importantly, as noted in humans, Tregs were enriched in PanIN, tumor edge, and tumor core relative to normal ducts (Supplemental Figure 8F). Comparison of lymphoid aggregates proximal to PanIN and PDAC revealed greater Treg density in PDAC-adjacent aggregates, but the number of aggregates was too small to yield statistical significance. Differential protein expression in CD8^+^ T cells between lesion types revealed lower PD1 expression in PanIN-adjacent CD8^+^ T cells (45 cells) than at PDAC tumor edges (158 cells) or tumor cores (204 cells) (Supplemental Figure 8H). This possibly reflects a more exhausted T cell phenotype, as with the human data.

As observed with the human data, we also found that in tiKPC mice (a) PanIN and PDAC recapitulate features of T cell accumulation relative to normal ducts, and (b) CD8^+^ T cells proximal to PanIN exhibit lower PD1 compared with PDAC. The lymphoid aggregates observed in mice also showed a trend toward increased Treg density in PDAC over PanIN, consistent with human PDAC. Taken together, these results suggest that these lymphoid aggregates become more immunosuppressive with progression to PDAC.

### Spatial transcriptomics of human biospecimens identifies markers in peritumoral TLSs.

**Figure 5:**
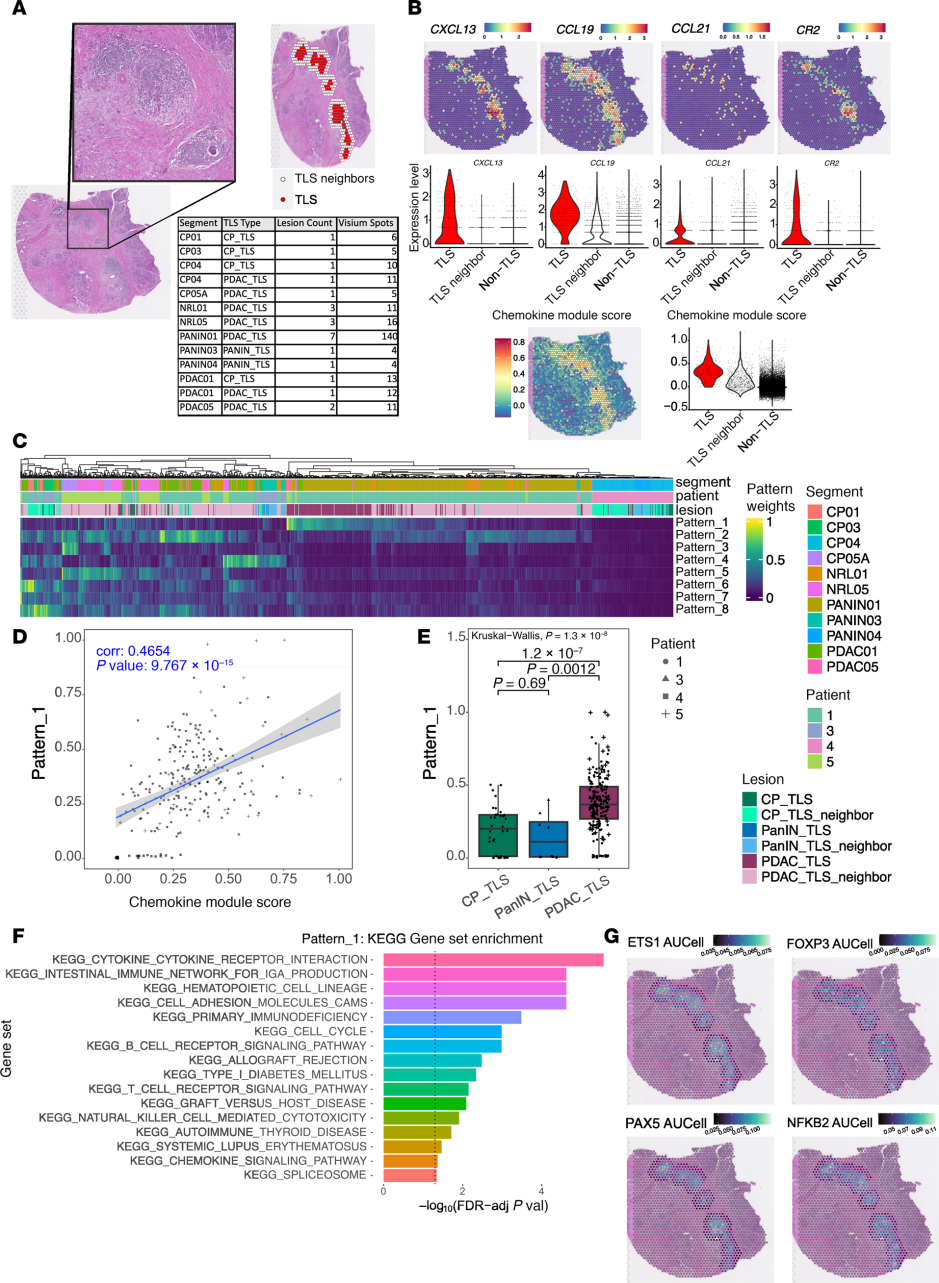
10× Genomics Visium spatial transcriptomics platform recapitulates TLS signatures adjacent to PDAC. (**A**) TLS annotation on H&E-stained segments by a pathologist followed by automated annotation of all Visium spots within 2 spots of the boundaries of any TLS as TLS neighbor. TLS types were then assigned by the pathologist based on their proximity to chronic pancreatitis (CP_TLS), pancreatic intraductal neoplasms (PANIN_TLS), or pancreatic ductal adenocarcinoma (PDAC_TLS). (**B**) Expression of *CXCL13*, *CCL19*, *CCL21*, and *CR2* (CD21), and a module score based on expression of chemokine-encoding genes in TLS (red), TLS_neighbor (white), and non-TLS (gray) Visium spots as violin plots and representative spatial expression plots showing segment PANIN01. (**C**) Heatmap of pattern weights in each Visium spot for the 8 patterns learned by CoGAPS on the TLS and TLS neighbor spots. (**D**) Scatter plot of association between the chemokine module score on the *x* axis and Pattern_1 weight on the *y* axis. Points are overlaid with the trend line of the linear regression of Pattern_1 weight on chemokine module score and calculated Pearson’s correlation coefficient. (**E**) Box plot of Pattern_1 weight comparing CP_TLS (dark green), PANIN_TLS (dark blue), and PDAC_TLS (dark red). Shapes of each data point correspond to the patient from whom the Visium spot originated. Weights were compared across all groups using a Kruskal-Wallis test (*P* = 1.3 × 10^–8^). Bars are annotated with *P* values of pairwise comparisons by Wilcoxon’s rank-sum test. (**F**) Waterfall plot of gene set enrichment analysis of KEGG gene set in genes ranked by pattern marker statistic for Pattern_1. Gene sets with significant enrichment (FDR-adjusted *P* value < 0.05) are shown. Enriched gene sets are ordered by –log_10_(FDR-adjusted *P* value). (**G**) Transcription factor activity scores of *ETS1*, *FOXP3*, *PAX5*, and *NFKB2* inferred by SCENIC gene regulatory network inference and quantified by AUCell overlayed on segment PANIN01.

We employed the 10× Genomics Visium to investigate tissue collected from FFPE blocks of TLSs found in proximity to PanINs and PDAC (24). Nineteen of the 20 segments from 5 patients had sufficient read depth and unsupervised clustering that aligned with histologic features for subsequent analysis. Six segments across 4 patients contained low-grade PanIN and one segment contained high-grade PanIN, per pathologists. All lesions were categorized as “PanIN” in subsequent analysis, regardless of grade, due to high-grade PanIN being represented by a single sample (Supplemental Figure 9 and Supplemental Table 9). Twenty-four TLSs were identified within 250 μm of CP (4), PanIN (2), or peritumoral (18) (Figure 5A). Relative to surrounding spots and non-TLS tissue, TLSs exhibited increased expression of chemokine-encoding genes *CXCL13*, *CCL19*, and *CCL21*; the follicular dendritic cell and germinal center–associated B cell marker *CR2* (CD21); and a 12-chemokine-encoding gene signature that identified ectopic lymph node–like structures in microarray data from primary colorectal carcinoma (25) (Figure 5B).

TLSs associated with PanINs were rare, occupying a small area compared with PDAC TLSs, yielding few representative Visium spots for robust statistics. We thus employed non-negative factorization of gene expression data from the TLS spots (Figure 5A, red dots) and neighboring regions within 2 TLS spots (Figure 5A, white spots) using CoGAPS (26). CoGAPS learned 8 transcriptional patterns. Patterns 2, 3, and 5–8 were specific to spots in stromal regions in proximity to TLS, and Pattern 4 was enhanced in regions of acinar tissue (Figure 5C and Supplemental Figure 10A). Pattern 1 showed the greatest association with TLSs. Overlay of Pattern 1 weights on TLS images showed high pattern weights in centers of TLSs, with a gradient of declining weights to the TLS periphery. Pattern 1 weights for each spot correlated with the 12-chemokine module score computed for each spot (Pearson’s coefficient 0.4654, *P* = 9.767 × 10^–15^) (Figure 5D), recapitulating the existence of mature TLSs adjacent to PDAC using IMC. Pattern 1 weights were greater in PDAC-proximal TLSs compared with PanIN-proximal TLSs (*P* = 0.0012) or CP-proximal TLSs (*P* = 1.2 × 10^–7^) (Figure 5E). This latter finding provides further evidence for the differences in maturity and functionality in PanIN- versus PDAC-associated TLSs, as with IMC.

We captured marker genes for each pattern using a novel pattern marker statistic, which assigns each gene as marker of patterns associated with higher fractional expression, allowing a gene to be a marker of multiple latent patterns identified by CoGAPS by considering high, medium, or low fractional expression of each across the dataset. Pattern marker scores for each pattern were used for gene set enrichment analysis using the Kyoto Encyclopedia of Genes and Genomes (KEGG) (27–29). Pattern 1 showed high representation of pathways, including B and T cell receptor signaling, and TLS maturation pathways, including cytokine-cytokine receptor interactions, cell cycle processes after antigen recognition, and costimulatory receptor engagement (Figure 5F). Patterns 2, 3, and 6 were enriched for pathways associated with extracellular matrix interactions and cell adhesion. Pattern 4 showed enhancement of metabolic pathways associated with acinar cells. Enrichment of autoimmune processes and cytokine signaling in Pattern 8 suggest inflammatory signals from cells in the stroma. No KEGG pathways were enriched among marker genes for Patterns 5 or 7 (Supplemental Figure 10B). The association of Pattern 1 spot weights in PDAC-associated compared with human PanIN-associated TLSs is consistent with IMC showing that PanIN TLSs are less mature.

We used pySCENIC (30, 31) to infer transcription factor activity in the same Visium spots from TLSs and their neighbors. Transcription factor genes with activities displaying high correlation with Pattern 1 revealed TLS cores with high representation of transcription factors associated with T and B cell development, namely *ETS1* and *PAX5*, as well as *NFKB2*, encoding a transcription factor with broad roles in immune regulation (32–34) (Figure 5G). These findings of transcription factor activity associated with lymphocyte development and activation within TLS centers is supported by the observed organization of a B cell germinal center bounded by T cells in PDAC-associated TLSs using IMC (Figure 3B). We also found that *FOXP3*, a Treg transcriptional marker, showed sparse detection (Supplemental Figure 11), likely due to dropout (gene not captured by the spatial probes) of these transcripts, rather than the paucity of Tregs (based on their proteomic detection). When assessing the activity of *FOXP3* as a transcription factor based on expression of genes in its regulon (Figure 5E), activity was enhanced around the periphery of TLSs in contact with tumor stroma. This observation follows the expected distribution of T cells in a mature TLS and suggests that the Tregs found to be more abundant in PDAC-associated TLSs compared with PanIN TLSs may play a role in hindering the efflux of mature lymphocytes from the TLS into the tumor.

## Discussion

We present a rigorous analysis of the localization and characterization of lymphoid aggregates that are associated with treatment-naive PDACs, PanINs, and CP in resected specimens from the same patient. We also report the existence of organized lymphoid structures with distinct structural and functional characteristics that differ between those adjacent to PDAC, PanIN, or CP and were not observed in normal ductal tissue. Furthermore, PanINs were clonally distinct from patient-matched tumor samples; thus, the immune microenvironment of each PanIN was genetically distinct from that of associated PDAC. We observed genetic heterogeneity among PanIN and PDAC lesions present in the same tissue block in agreement with Braxton et al. (14). We also associated features of the immune response to PDAC progression using spatial proteomics and transcriptomics. Observed lymphoid structures differed between PanINs and PDACs. While PanINs attracted less organized T and B cell aggregates, PDAC invasive fronts were associated with mature TLSs with defined germinal centers and follicular cells. Lymphoid structures in mice were more similar to the lymphoid aggregates observed in human PanINs rather than mature TLSs observed adjacent to PDACs, likely explained both by the shorter duration of antigen exposure and fewer available antigens to react against (35).

We posit that the immune cell composition of PanINs is more favorable for immune-based interception. First, IMC revealed fewer Tregs in PanIN, while densities in tumor regions were higher. These Tregs were in closer proximity to cytotoxic CD8^+^ T cells within the tumor compared with PanINs, suggesting increased immunosuppression in tumors. Second, we found that the ratio of cytotoxic CD8^+^ T cells to Tregs was higher in PanINs compared with tumors. Finally, PanINs had a lower exhausted T cell density compared with tumor. A caveat is that, while our analysis was limited to low-grade PanINs, high-grade lesions may display a more evolved immunosuppressive microenvironment but are rarely identified in isolation from PDAC.

Recent landmark studies have reported on the surprisingly high PanIN frequency in non-PDAC autopsy specimens, setting the stage for identifying factors in the PCE that may restrain or promote PDAC progression. Our observations are consistent with Carpenter et al. in that CD4^+^ (and not CD8^+^) T cells are enriched in PanINs. In contrast with Carpenter et al., we observed Treg enrichment in PanINs — a discrepancy that we attribute to the difference between spontaneous PanINs from non-PDAC-affected individuals versus PanINs associated with PDAC. This brings into question whether Treg-enriched PanINs are more likely to progress to PDAC, as in the case of our murine studies. Furthermore, given that there are no reports of TLSs in Carpenter et al., it behooves us to determine whether TLSs occur sporadically alongside PanINs in non-PDAC-affected individuals.

While immune exclusion is frequently described in the context of PDACs, there is less data regarding the composition of immune populations restricted to the tumor border. We posit that, among the relatively dense immune microenvironment restricted to just outside the tumor, T cells are educated by TLSs to recognize specific antigens. However, these T cells are unable to infiltrate into the tumor due to the dense stroma. TCR sequencing should provide information on T cell clonality within the tumor compared to peritumoral region; this was attempted, albeit without success due to sparse T cells infiltrating the tumor.

The distinctive features between immature and mature TLSs have previously been described, with immature TLSs comprising CD21^+^CD23^+^ B cells, but lacking germinal center formation (22, 23). Interestingly, as in precancer lesions of liver (36) and breast cancers (37), we find immature TLSs associated with both human and mouse PanIN lesions. However, we did not find mature TLSs in proximity to PanINs, contrasting the mature TLS phenotype described in intrapapillary mucinous neoplasms (IPMNs), another type of PDAC precancer (38). In the mouse, immature, but not mature, TLSs, were associated with both PanIN and PDAC. That murine PanINs developed temporally prior to PDAC supports the notion that PanIN themselves induce inflammation that results in immature TLSs, independent of PDAC. Notwithstanding our inability to temporally profile human tissue, our mouse study provides an explanation that immature TLSs in human PanIN lesions likely develop independently of coexisting PDAC. However, mature TLSs have not been identified in the tiKPC mouse.

**Figure 6:**
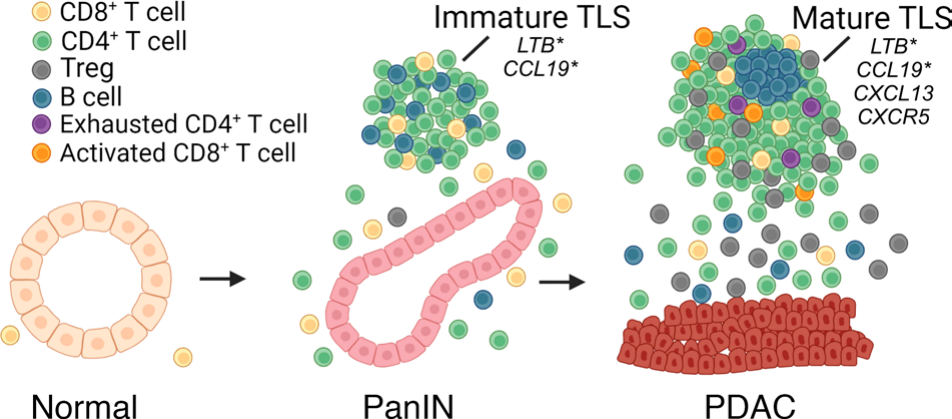
Proposed model of TLS formation from normal to PanIN to PDAC progression. Immune cells (T and B cells) are recruited to early developing PanIN lesions, demonstrating early organization into immature tertiary lymphoid structures (TLSs). In PDAC, these TLSs are organized further, often presenting with a distinct zone of B cells forming a germinal center surrounded by T cells and B cells. This T cell population within the PDAC-associated TLSs is also composed of increased immunosuppressive Tregs, exhausted CD4^+^ T cells, and activated CD8^+^ T cells. Transcriptional signatures indicate *LTB* and *CCL19* overlapping with a PanIN-adjacent TLS, whereas PDAC-adjacent TLSs share these signatures plus *CXCL13* and *CXCR5*. *Indicates shared genes.

The low levels of proliferation and activated T cell markers in immature TLSs in PanIN indicate limited functionality. Furthermore, our exploration of the transcriptomic differences between PanIN-associated immature and mature TLSs (found outside the tumor margin) showed a correlation with B cell development transcription factors and protein expression, except *NFKB2* activity, which was not included in the IMC panel. While the chemokine-related genes *LTB*, *CCL19*, *CXCL13*, and *CXCR5* were expressed in PDAC-associated mature TLSs, only 2, namely *LTB* and *CCL19*, overlapped with PanIN-associated immature TLSs (Figure 6). This gain of chemokine-related genes in PDAC may thus reflect an evolving microenvironment that begins with immature TLSs associated with PanINs and progresses slowly to mature, tumor-excluded TLSs adjacent to the PDAC. Alternatively, low levels of tumor antigen in PanINs may prevent maturation of the associated immature TLSs. This may also account for the observation of mature TLSs in the context of IPMN, which are typically much larger than PanIN lesions, and are radiographically visible (38). To this point, *CXCL13* which was expressed by the mature TLS adjacent to tumors was associated with a signature identifying neoantigen-reactive T cells (39), suggesting that PDAC-associated mature TLSs have the capacity to educate tumor-specific T cells similar to TLSs found in autoimmune diseases (40). TCR and BCR sequencing of PanIN- versus PDAC-associated TLSs should provide further clues on antigen recognition by the respective cellular components. Additionally, while we identified mature TLSs in the periphery of PDACs, the functionality of any activated T cells within these TLSs is likely mitigated by immunosuppressive, exhausted, and senescent T cells populating the area.

Multiple factors in the evolving PCE and TME may account for TLS development. First, the stroma acts as a physical barrier to T cell ingress (41). Second, data from a mouse melanoma model suggest that cancer-associated fibroblasts induce variable levels of lymphoid-inducing signals (42) to contribute to TLS evolution. Finally, a proinflammatory stroma can upregulate *CXCL13* expression, suggesting that stromal elements might affect mature TLS formation (43). This finding is consistent with the absence of transcriptional upregulation of *CXCL13* in PanIN-associated TLSs.

Therapeutically, the decade or more long window for human PanINs to progress to PDAC provides an exceptional opportunity to intercept cancer progression in high-risk individuals. For this, gaining a deeper understanding of early events associated with PDAC progression becomes imperative. In this respect, the tiKPC mouse is likely to remain a vital tool not only for mechanistic studies into the quantity, function, and localization of immune cell subtypes and the immune landscape in both PanINs and PDAC lesions, but also as a platform for testing novel immune interception strategies that could propagate TLS maturation.

Exogenous antigens delivered in the form of vaccines may potentiate mature TLS development. HPV vaccines have resulted in mature TLS formation in regressing lesions in individuals with cervical intraepithelial neoplasia (44). Vaccine adjuvants, including TLR agonists (e.g., polyICLC, CpG, or STING agonists), can promote TLS maturation. The intratumoral injection of CXCL13 and CCL21 has led to TLS formation in PDAC models (45). Given their ability to deliver antigen and immune-modulating agents simultaneously, nucleic acid–based vaccines may be a versatile interception platform focused on enhancing TLS maturation (46). Nonetheless, potential toxicities must be carefully considered when treating individuals at a high risk.

In summary, our study comprises a small but rare cohort of patients with treatment-naive PDAC, complemented with a mouse model for temporal analyses. It represents an in-depth spatial characterization of the immune architecture of early precancer lesions in PDAC. PanIN lesions in the absence of associated PDAC lesions are not possible to obtain in humans, except through the exploration of autopsy samples. The rarity of this resected PDAC cohort arises from the need to have PanIN and PDAC in the same patient section, but with the 2 lesions at spatially distinct locations. Furthermore, our discovery of immature TLSs in PanIN lesions lends a major challenge — to interrogate immature TLSs to define the earliest tumor-specific T cell responses and identify mechanisms that promote T cell ingress and activation. The temporal sequence of PanIN-to-PDAC progression in the tiKPC mouse provides a unique way to achieve this goal.

## Methods

### Sex as a biological variable.

Our study examined male and female patients, and sex was not considered as a biological variable due to the small sample size. Our study examined male and female mice, and sex was not considered as a biological variable.

### Human tissue acquisition and selection of samples.

Our study examined male and female patients with PDAC (details in Supplemental Table 10). Five patients without perioperative chemotherapy with distinct PanIN lesions were identified by pathologist. Four patients had PDAC, and one with distal cholangiocarcinoma infiltrating the pancreas. The latter was included to study treatment-naive PanIN lesions not associated with PDAC. The microsatellite instability-high (MSI-H) specimen (Patient 3) did contribute 4 of the 11 PDAC-adjacent TLSs (Supplemental Table 8). For one patient, ROIs for PDAC, PanIN, or CP were not available; however, there was sufficient ROIs, including peritumoral regions, that also included TLSs.

Serial sections were cut on unstained SuperFrost Plus (Thermo Fisher Scientific) slides for H&E, IHC, or IMC. Sections were cut onto PEN membrane slides activated with 30 minutes of UV light for laser capture microdissection. Punches were cut onto 10× Genomics Visium slides for spatial transcriptomics (Supplemental Figure 1A). Each H&E section was annotated by a pathologist and ROIs were established as a reference for IMC data acquisition (Figure 1A). Sections were preferentially chosen for analysis if they had PDAC, PanIN, CP, and normal regions. Immune-rich areas, particularly within regions of tumor, were identified using CD3 immunostaining.

CD3 immunostaining was performed on FFPE sections on a Ventana Discovery Ultra autostainer (Roche Diagnostics). Briefly, following dewaxing and rehydration on board, epitope retrieval was performed using Ventana Ultra CC1 buffer (Roche Diagnostics, 6414575001) at 96°C for 60 minutes. Primary anti-CD3 antibody (Abcam, catalog ab16669, lot GR3262328-4; 1:200 dilution, 36°C, 60 minutes) was captured by an anti-rabbit HQ detection system (Roche Diagnostics, 7017936001 and 7017812001). This was followed by Chromomap DAB IHC detection kit (Roche Diagnostics, 5266645001), counterstaining with Mayer’s hematoxylin, dehydration, and mounting. All H&E and IHC sections were scanned using Hamamatsu Nanozoomer, and images visualized using NDP.view2.

### IMC acquisition and analysis.

Resected-pancreas slides were baked at 60°C for 2 hours, dewaxed in histological grade xylene, and rehydrated in a descending alcohol gradient. The slides were incubated in Antigen Retrieval Agent (pH 9) (Agilent, S2367) at 96°C for 1 hour and blocked with 3% bovine serum albumin (BSA) in Maxpar PBS (Standard BioTools, 201058) at room temperature for 45 minutes. Antibody cocktails for human and mouse tissue sections (Supplemental Tables 2 and 3) were used to stain slides at 4°C overnight. Custom antibodies were conjugated in-house, diluted to 0.25 mg/mL to 0.5 mg/mL, and titrated empirically. Cell-ID Intercalator-Ir (Standard BioTools, 201192A) was diluted at 1:400 in Maxpar PBS and used for DNA labelling. Ruthenium tetroxide 0.5% aqueous solution (Electron Microscopy Sciences, 20700-05) was diluted at 1:2000 in Maxpar PBS and used as a counterstain. Images were acquired by Hyperion Imaging System (Standard BioTools), and representative images generated through MCD Viewer (Standard BioTools).

Images were segmented for analysis using nuclear (Ir^191^ and Ir^193^) and plasma membrane staining (IMC Segmentation Kit, Standard BioTools) (47). Twenty images were used to assign pixel classifications and establish predictions in Ilastik (48), which was used to segment images using CellProfiler (v4.2.4) (49). Each segmented image was further subdivided into increasingly strict regions using histoCAT (21) (based on the features found in each image) (Figure 1C). For example, a 1 mm × 1 mm region, which included a PanIN lesion and surrounding acinar tissue, was further subdivided into a region that included just the PanIN plus a 200-μm region beyond the edge of the PanIN epithelial cells (Supplemental Figure 1B). This approach restricted our immune cell analysis to the PanIN lesion while excluding the surrounding tissue. In contrast, several PDAC regions were not further subdivided, as the entire ablated region was composed entirely of intratumoral cells.

For the human dataset, each individual cell (both TLS and non-TLS regions) were clustered at a resolution of 50 clusters using FlowSOM (https://www.flowjo.com/exchange/plugin/flowsom). Annotations were assigned using the relative expression of marker panel (Figure 1C). For mouse, cells were clustered using Rphenograph v0.99.1 at *k* = 30 clustering resolution. Clusters with elevated CD45 expression were subjected to further clustering at *k* = 30 resolution, which were annotated by cell type based on characteristic protein markers. Density of cell types was determined by dividing the number of cells detected per cluster by the area of tissue analyzed (Supplemental Tables 2 and 4). Box-and-whisker plots were generated in R v3.6.3 (https://cran.r-project.org) using ggplot2. Hinges of the box correspond to the 25th percentile (bottom) and 75th percentile (top) of plotted data, with the center line denoting the median. Whiskers extend to the largest value plotted no further than 1.5 times the interquartile range. *P* values were derived from Wilcoxon’s rank-sum tests of cell density per area and were not adjusted for multiple test correction due to the low number of tests.

Interaction networks were generated by measuring the shortest distance between all cells computationally (utilizing *x* and *y* coordinates generated by the segmentation process) and depicting the relative distance between each cell type based on average shortest distances. Clusters were excluded from the analysis if they consisted of less than 1% of the total counts.

Top neighbor analysis was performed by compiling the top 2 neighbors per cell per cluster using CellProfiler (https://cellprofiler.org). A heatmap was generated to display the aggregated data. A cluster was excluded from the heatmap if it consisted of less than 1% of the overall counts. Normalized relationships were generated by dividing each matrix by the largest cell count per region type.

Representative images were prepared using MCD Viewer, overlaying multiple stains and adjusting the threshold to minimize background. These were then exported as 16-bit images into GIMP with minimal noise reduction (level 2).

### Spatial transcriptomics data generation.

Sample preparation followed manufacturer’s protocol for Visium FFPE (10× Genomics) using a Visium Spatial Gene Expression Slide Kit (10× Genomics, 1000185), Visium FFPE Reagent Kit (10× Genomics, 1000362), Visium Human Transcriptome Probe Kit (10× Genomics, 1000364), and Dual Index Kit TS Set A (10× Genomics, 1000251). Four tissue segments each were collected from 5 surgical specimens by scoring the paraffin block with a skin punch (5 mm) before sectioning. Segments were placed on 10× Genomics Visium slides within the 4 fields of maximum 6 × 6 mm size, with each slide containing normal, PDAC, PanIN, and CP from the same surgical specimen. Segments were deparaffinized, stained with H&E, and scanned using a Nanozoomer scanner (Hamamatsu) at 40×. Human probe hybridization was performed overnight at 50°C using the Visium Human Transcriptome Probe Set v1.0. RNA was digested following probe ligation, and tissue permeabilized for release, capture, and extension of probes. Probes were captured for sequencing by oligo-d(T) capture. Sequencing libraries were prepared following the manufacturer’s instructions to extend probes as the template. All libraries were sequenced at a minimum depth of 50,000 reads per spot (minimum of ~250 million per sample) on a NovaSeq (Illumina). The Visium Human Transcriptome Probe Set v1.0 contains probes targeting 19,144 genes, which provided gene expression information on 17,943 genes after filtering for probes with off-target activity.

### Image-based tissue type annotation with CODA.

Seven microanatomical components of human pancreatic tissue were multilabelled with a semantic segmentation workflow using CODA (50), per Bell et al. (24). A neural network trained on 25 annotated examples of pancreatic tissue was used to annotate each pixel of the spatial image as islets of Langerhans, normal ductal epithelium, vasculature, fat, acinar tissue, collagen, PDAC, PanIN, or non-tissue. Nuclear coordinates were generated via the detection of 2D hematoxylin intensity peaks. The low-resolution tissue image of each segment provided by Space Ranger was registered to the high-resolution tissue image using the fiducial markers on the spatial transcriptomics slide. Visium spot coordinates were registered in the high-resolution image annotated by tissue types and cellularity was calculated within 25 mm of each Visium coordinate. Tissue composition was determined by analyzing the percentage of each classified tissue type with each spot. Cellular identity was estimated by determining the microanatomical label at each coordinate where a nucleus was detected. Spots were labeled by the predominant tissue type if the spot comprised 70% or more of the same tissue type. Spots where “no-tissue” type made up 70% or more of the spot were annotated as “NA,” indicating mixtures of multiple tissue types. Spots annotated by the pathologist as “PanIN” were further delineated into “low-grade” or “high-grade” PanIN based on cell morphology. Where CODA annotations of PanIN did not agree with morphology, we deferred to the pathologist’s assessment for labeling.

### Spatial transcriptomics data analysis.

Space Ranger v1.3.1 (10× Genomics) was used to demultiplex sequencing data, convert FASTQ files of spot barcodes and transcript reads, align barcodes to the spatial image, and generate read count matrices. Subsequent data processing and analysis was conducted in R v4.2.0 using Seurat v4.1.1 (51). Expression data for each tissue segment was loaded into R and underwent initial visualization of UMI counts and detected gene number per spot to assess sample quality. Read count normalization per segment used the SCTransform function with the negative binomial method followed by clustering of cells using Leiden clustering with Leidenalg v0.8.0 (52). Using Loupe Browser v6.4.0 (10× Genomics), spots from pieces of tissue that had broken apart from their native context and spots where tissue had folded onto itself were annotated for export of spot barcodes and removed from subsequent analysis. All spots annotated as “fat” by CODA were removed from subsequent analysis to exclude spots where the high density of probes from acinar tissue may bleed into neighboring RNA-poor regions of adipocytes. The segment PANIN04 was not included in subsequent analysis, as it had very low UMI detection per spot (median 171 UMI per spot) and Leiden clusters did not follow tissue morphology.

Upon filtering spots and segments based on quality and tissue types, segments were aggregated into Seurat objects for each patient that were processed and sequenced together. Expression counts underwent normalization and scaling using SCTransform for each patient. Spatially variable features were identified using the FindSpatiallyVariableFeatures function and used for principal component analysis. The first 25 principal components for each patient were used to identify spot neighborhoods, calculate UMAP embeddings for spots in each patient, and identify Leiden clusters. The AddModuleScore function was used to identify increased coexpression of genes associated with classical PDAC, basal PDAC, cancer stem cells (24), and a 12-chemokine gene signature that delineates microarray data from solid tumors containing TLSs (25).

TLSs were identified within tissue segments by the pathologist’s review of H&E images for TLSs within 250 μm of PDAC, PanIN, or CP on sequential slides from the surgical specimens. Visium spots corresponding to the TLSs were picked using Loupe Browser where cell barcodes were exported for annotation of the spots in Seurat. Visium spots from neighboring regions surrounding TLSs were selected automatically using STUtillity v1.1.0 (53) where STUtility objects were created using the same Space Ranger outputs as the Seurat objects and cell barcodes for TLS regions. The RegionNeighbors function was used to identify all spots within 2 Visium spots (~150 mm) of TLS boundaries. Spots within TLSs and the 2-spot neighboring regions underwent non-negative matrix factorization using CoGAPS v3.14.0 (26). CoGAPS was run on log_2_-transformed counts with +1 pseudo counts to learn 10 patterns with 50,000 iterations on “genome-wide” distributed CoGAPS mode. Sparsity parameters were α = 0.01 and maxGibbsMass = 100. Distributed parameters were nSets = 16, cut = 10, minNS = 8, and maxNS = 24.

Spatial plots and violin plots of TLS marker genes and the chemokine module score were generated with the SpatialFeaturePlot function and VlnPlot functions, respectively. TLS annotations were plotted with SpatialDimPlot. Heatmaps of pattern weights stored as sampleFactors in the results from CoGAPS were generated with ComplexHeatmap v2.12.0 (54). Pattern weights in TLSs were compared across PDAC-, PanIN-, and CP-associated TLSs using the Kruskal-Wallis test across all groups and Wilcoxon’s rank-sum test for pairwise comparisons (R stats v4.2.0). Pearson’s correlation between the chemokine gene signature module score and CoGAPS Pattern 1, showing association with TLS cores, was calculated with the cor.test function and goodness of fit *R*^2^ values were calculated using linear regression with the lm function.

Pattern markers were assigned based on an approach innovating upon the method of Stein-O’Brien et al. (55). In brief, genes from the expression matrix (D) were categorized into distributions based on low, medium, or high fractional expression of the estimated reconstruction of the expression matrix (D′). Genes that were identified as outliers for the expected expression distribution in a given pattern were annotated as a marker of that pattern. Gene scores as a pattern marker from the outlier analysis were used to rank genes for GSEA using the R package fgsea v1.22.0, with gene sets from KEGG (27–29) obtained through the Molecular Signatures Database (MSigDB) (56, 57) using msigdbr (v7.5.1). Transcription factor activity scores were quantified for TLS spots and neighboring spots within 2 spots using pySCENIC (30, 31) v0.11.0 with refseq-r80 reference for transcription factor–encoding genes and rankings for transcription factor binding sites within 500 bp up to 100 bp down and 10 kb up and 10 kb down of transcription start sites of genes in the hg38 reference genome. Motifs reference v9 was used sourced from the Aerts lab cisTarget database (https://resources.aertslab.org/cistarget/).

### Laser capture microdissection for whole-exome sequencing preparation and analysis.

Serial FFPE tissue sections (10 μm^2^) were cut onto UV-activated (30-minute exposure to UV light) PEN membrane slides. Tissue sections were stored for up to 2 weeks at –20°C before microdissection. Slides were heated at 40°C for 20 minutes, deparaffinized in fresh xylene for 5 minutes twice, then rinsed in diH_2_O 6 times. Slides were rinsed in 100%, 95%, and 70% ethanol each for 1 minute and stained with 50% hematoxylin for 1 minute, rinsed for 1 minute in diH_2_O, and stained with 50% eosin for 2 seconds. The slides were rinsed in 70%, 95%, and 100% ethanol each for 1 minute. The stained slides were air dried and laser capture microdissection was performed using LMD 7000 system (Leica). The adjacent H&E sections were used as reference for areas that would be microdissected (based on annotations by pathologist). PanIN regions were defined by the PanIN lesion itself plus a 250 μm surrounding edge. Large areas were taken within the tumor and CP regions, and normal tissue to maximize the DNA collected (representative images in Supplemental Figure 12). Ten (10 μm^2^ thick) sections were cut per region per patient to collect enough DNA to submit for whole-exome sequencing. DNA was extracted using a QIAamp DNA FFPE Tissue kit (Qiagen, 56404).

Somatic mutations were called for each PanIN, PDAC, or CP sample with a matched normal using the GATK toolkit (v4.3.0.0) following best practice pipeline (58). A panel of normals (PONs) was generated by running Mutect2 on each normal sample using the hg38 human reference genome with the max-mnp-distance set to zero, and then using the GenomicsDBImport function followed by CreateSomaticPanelOfNormals function. After the PONs were created, somatic mutations for each sample of interest were called using Mutect2. A contamination table for each sample was created using GetPileupSummaries followed by CalculateContamination. Artifact priors were calculated using the LearnReadOrientation function. Variants were filtered using the contamination table and artifact priors. Variant annotation was done using OpenCRAVAT (v2.2.7) and variants, namely frameshift insertion, frameshift deletion, in-frame deletion, in-frame insertion, missense variant, splice site variant, stop gain, stop loss, and synonymous variant were selected (59). Selected variants also needed to have at least 30× coverage in both tumor and matched normal samples with at least 6 reads in the tumor sample supporting the variant. Finally, a visual inspection was performed to remove a variant if (a) all reads supporting the variant were from the same strand, (b) VAF of the variant was 100%, (c) supporting reads were towards the end of the read, (d) reads were low quality, and/or (e) supporting reads had the same start and end position. Mutations for each patient were visualized using UpSetR (v1.4.0) (60). One patient had insufficient DNA from the normal sample to allow for curation of the mutation calls specific to PanIN and PDAC. Another patient was diagnosed with distal cholangiocarcinoma so the comparison between PanIN- and PDAC-specific mutations was not considered feasible.

### tiKPC murine model and tissue acquisition.

Tamoxifen-inducible *Kras*^LSL-G12D^; *p53*^LoxP^; *Pdx1*–CreER (tiKPC) mice were purchased from The Jackson Laboratory (stock 032429) and bred in-house to generate the *Pdx1*-*CreEr^Tg/Tg^*;*Trp53^fl/fl^*;*Kras*^G12D/–^ genotype. tiKPC mice at 8–10 weeks of age were induced with intraperitoneal injections of 100 μL tamoxifen (20 mg/mL) for 5 days (1 g tamoxifen [Sigma-Aldrich, T5648] plus 2.5 mL pure ethanol, vortexed for 5 minutes). Fifty milliliters prewarmed (55°C) sunflower oil (Sigma-Aldrich, S5007) was added to the tamoxifen/ethanol mixture in an Erlenmeyer flask, and the solution was dissolved in a shaking water bath at 175 rpm, 55°C for up to 1 hour. The dissolved solution was aliquoted in amber Eppendorf tubes and stored at –20°C. On the day(s) of injection, aliquots were warmed in a heating block for 10 minutes until fully thawed.

Pancreatic tissue and any pancreatic tumors were dissected from each mouse at time points predetermined before induction was initiated. Tissue was sandwiched between 3 surgical sponges (2 on bottom, 1 on top) (Thermo Fisher Scientific, 22-038-221) during fixation in 10% Neutral Buffered Formalin (LabChem, VT450D) for up to 24 hours. This maximized the surface area exposure of the tissue and ensured consistent thickness across all samples.

FFPE tissue sections (5 μm thick) were cut, and 6 H&E sections produced for each mouse to explore the entire pancreas. Nine serial sections were cut onto SuperFrost Plus slides for long-term storage at –80°C. A pathologist defined the ROIs as a reference for IMC data acquisition. H&E tissue sections were labeled as Tumor Edge, Tumor Core, PanIN, Lymphoid Aggregate, or normal, and were scanned at 40× resolution using Hamamatsu Nanozoomer with images being visualized by NDP.view2.

### Statistics.

A *P* value of less than 0.05 was considered significant. Multiple test correction for GSEA tests of marker gene scores in learned pattern weights was conducted using Benjamini-Hochberg false-discovery rate adjustment. Box-and-whisker plots denote median, interquartile range, and 5th and 95th deciles. Data points are overlaid as shapes representing the patient from which they were measured. Regions subjected to IMC were excluded from analysis if cholangiocarcinoma was present.

### Study approval.

All human specimens were obtained with patient consent and approval by the Institutional Review Board (NA_00001584). All mouse experiments were conducted with approval from the Johns Hopkins University Animal Care and Use Committee (Mo22M98).

### Data availability.

Spatial transcriptomics and IMC datasets are available from the NCBI Gene Expression Omnibus (GEO GSE254829 and GSE294669). Human and mouse data are available from https://doi.org/10.5281/zenodo.8336719 and https://doi.org/10.5281/zenodo.14751512, respectively. Code for data analysis is available from https://github.com/FertigLab/Human_PanIN_Spatial_Analysis Values used to generate figures are available in the Supporting Data Values file.

## Author contributions

MRL, JTM, LTK, EDT, EMJ, EJF, WJH, and NZ contributed to study design. MRL, JTM, LTK, SMS, ALH, EMC, EDT, EMJ, EJF, WJH, and NZ contributed to experiment design. MRL, JTM, LTK, SDH, SMS, BB, GL, ALH, EMC, XY, LA, ALK, AF, DW, PHW, EDT, WJH, and NZ contributed to data acquisition. EDT, JWL, and RAA assessed tissue specimens. MRL, JTM, LTK, SR, ALH, SMS, SG, JWL, YZL, RK, AG, ALK, AF, DW, PHW, AD, JWL, TDA, JWZ, LDW, RAA, EDT, EMJ, EJF, WJH, and NZ contributed to data interpretation and analysis. MRL, JTM, SMS, JWL, EMJ, EJF, WJH, and NZ contributed to manuscript preparation. MRL, JTM, LTK, ALH, SDH, SMS, SG, BB, GL, AH, EMC, XY, LA, JWL, YZL, RK, AG, ALK, AF, DW, PHW, AD, JWL, TDA, NSA, JWZ, LDW, RAA, EDT, EMJ, EJF, WJH, and NZ contributed to manuscript review. MRL led study design, experiment design, analysis of human spatial proteomics data, and writing of the manuscript’s first draft. JTM led analysis of transcriptomics data, analysis of mouse proteomics data, and addressing comments received upon peer review.

## Supplementary Material

Supplemental data

Supporting data values

## Version 1 06/26/2025

In-Press Preview

## Version 2 08/08/2025

Electronic publication
