# Supplementary material for: Spatial proteomics and transcriptomics reveal early immune cell organization in pancreatic intraepithelial neoplasia
Source: JCI Insight. 2025 Jun 26;10(15):e191595. doi: 10.1172/jci.insight.191595 (PMC12333942; doi:10.1172/jci.insight.191595)
Supplement: Supplemental data [file jciinsight-10-191595-s044.pdf]

Supplemental Materials

Supplemental Figure 1: A) Serial sections were cut from blocks chosen by an expert pathologist containing 4 regions of interest (normal, PanIN, PDAC and chronic pancreatitis) for analysis using imaging mass cytometry (IMC), immunohistochemistry (IHC), Visium spatial transcriptomics, and laser capture microdissection (LCM) for DNA isolation and subsequent WES analysis. **B**) The step-wise process for staining, segmenting and pre-processing images collected for the imaging mass cytometry analysis. Quality control of segmentation and cell type identities was assured using a matrix of individual cell IDs, their x,y coordinates, and their corresponding phenotypes based on the immune markers (see Supplemental Table 1).

Supplemental Figure 2: Representative IHC staining for CD3+ cells in the same tumor section where there are T cell rich regions but also relatively immune-poor regions.

Supplemental Figure 3: Representative IMC images from 2 patients of normal, PanIN, PDAC, and CP regions demonstrating epithelial tissue specific markers (CK – cytokeratin, PM – plasma membrane, VIM – vimentin, KI-67, SMA – smooth muscle actin, DNA). Scale bars: 100 μm.

Supplemental Figure 4: **A**) Contribution of cells from each patient to each region of interest. **B**) Principal component analysis of all IMC samples in the dataset.

Supplemental Figure 5: Interaction network of normal, CP, PanIN and PDAC regions of all cell types that consisted of at least 1% of the total cells analyzed. Each node indicates a specific cell type colored by their identity. The node size correlates with the relative abundance of the given cell type in the region of interest. The thickness of the edges connecting cell type nodes correlates with proximity.

Supplemental Figure 6: A) Quantity of lymphoid cells per mm2 of tissue analyzed. Each color in the bar indicates the percentage of the total cells that comprise of each specific cell type. Statistics comparing percent composition between within tumor and tumor adjacent regions were determined using a Wilcoxon rank sum test. **B**) Interaction network of the peri-tumoral region. All cell types that consisted of at least 1% of the total cells analyzed. A node indicates a specific cell type colored by their identity. The node size correlates with the relative abundance of the given cell type in the region of interest. The thickness of the edges connecting cell type nodes correlates with proximity.

Supplemental Figure 7: Representative images depicting the distance measured by pathologist (E.D.T.) from either PDAC, CP or PanIN regions.

Supplemental Figure 8: Tamoxifen-inducible KPC (tiKPC) mouse model recapitulates spatial features of T cell response to PanIN and PDAC. **A**) Graphical outline of the tiKPC mouse genotype and treatment regimen consisting of daily tamoxifen doses for five days to induce carcinogenesis and sacrifice at 10 to 16 weeks post-induction for tissue collection. **B**) Mean protein expression of immune cell markers among labeled cell types in multiplex immunohistochemical (IMC) profiling of mouse pancreases. Cell types were annotated based on marker gene expression following hierarchical clustering. Lymphoid aggregates are circled in green. **C**) Representative tissue images of T cell markers in normal duct, PanIN, tumor edge, tumor core, and lymphoid aggregates. Hematoxylin and eosin-stained tissues are shown alongside IMC-quantified expression of CD3 (white), CD8 (yellow), FOXP3 (red), and nuclear stain (blue). Lymphoid aggregates are circled in green. **D-F**) Cell type density comparisons across tissue regions in cells/mm2. Densities of all T cells (**D**), CD4 T regs (**E**), and CD8 T cells (**F**) are compared across lesion types. **G**) Density of T regs in lymphoid aggregates proximal to PanIN compared to PDAC.

H) Violin plots of normalized PD1 protein expression in CD8 T cells across lesion types. Violins are overlayed with points representing expression in individual cell. Densities were calculated using the number cells per mm^2 of each ROI and compared by Wilcoxon rank sum test. Normalized protein expression was compared by Wilcoxon rank sum test, and calculated p-values were false discovery rate-adjusted accounting for 33 tested proteins. P-value <= 0.0001, 0.001, 0.01, 0.05, or > 0.05 are denoted in the plots with the respective symbols ****, ***, **, *, or ns.

Supplemental Figure 9: Representative images of PanIN lesions in Visium samples. **A, B)** H&E images of sample PANIN03 containing high-grade PanIN (**A**) and CP05B containing low-grade PanIN (**B**). **C, D**) CODA tissue annotation of PANIN03 (**C**) and CP05B (**D**) images used for spot tissue deconvolution. Colors correspond to PDAC (orange), PanIN (red), ductal epithelium (blue), islets of Langerhans (teal), smooth muscle (green), nerve (brown), lymph node (black), fat (yellow), acini (purple), collagen (pink). **E, F**) Magnified views of representative high-grade (**E**) and low-grade (**F**) PanIN lesions.

Supplemental Figure 10: Biological features associated with learned CoGAPS patterns. **A**) Representative spatial plots of pattern weights for patterns 2 through 7. **B**) Significantly enriched KEGG gene sets (FDR-adjusted p-value < 0.05) in CoGAPS patterns based on lists of genes ordered by CoGAPS pattern marker score.

Supplemental Figure 11: Normalized gene expression of FOXP3 across all Visium segments using SC transformation. Sparse expression of this characteristic transcription factor for T regulatory cell identity demonstrates the difficulty of detecting functional T cell states by individual marker gene expression.

Supplemental Figure 12: A, B) Representative images before and after laser capture microdissection (LCM) of specific regions of PanIN (**A**), PDAC, CP and normal tissue (**B**) for DNA extraction and whole exome sequencing.

Supplemental Table 1: KRAS mutations detected in laser capture microdissection samples of PDAC and PanIN.

Supplemental Table 1: KRAS mutations detected in laser capture microdissection samples of PDAC and PanIN.

Supplemental Table 2: Summary of imaging mass cytometry antibodies used to stain human pancreas tissue resections for this study.

Supplemental Table 3: Summary of imaging mass cytometry antibodies used to stain mouse tiKPC pancreas tissue resections for this study

Supplemental Table 4: Summary of the total area of tissue analyzed for each region. Sample number indicates the number of distinct regions analyzed per tissue section. The ROI indicates a more specific region within a sample. PanIN samples, for example, were divided so that only the immune cells were counted if they were within 200um from the epithelial cell edge of the lesion (See Figure 1C). The rest of the PanIN sample was indicated as "PanIN adjacent" in the metadata.

Supplemental Table 5: Summary of the total number of ROIs and total area of tissue that was analyzed per patient. These ROIs comprise the data described in Figure 2.

Supplemental Table 6: Summary of TLSs included in this IMC dataset. Maturity was determined by the presence of CD21 and CD23 staining within the defined TLS regions.

Supplemental Table 7: Summary of the total area of TLSs analyzed for each region. Sample number indicates the number of distinct TLSs per region included in this analysis. There were a grand total of 116 TLSs not analyzed in this dataset but found distributed throughout the whole tissue sections used for this cohort of data.

Supplemental Table 8: Summary of the total area and quantity of TLSs analyzed for each patient in the study.

Supplemental Table 9: Counts of visium spots containing low-grade (LG) PanIN, high-grade (HG) PanIN, normal duct, and PDAC in each Visium segment based on CODA annotation with subsequent pathologist verification. Lesion columns quantify the number of distinct PanIN lesions found in the segment.

Supplemental Table 10: Clinical and Pathological Features

Supplemental Figure 1

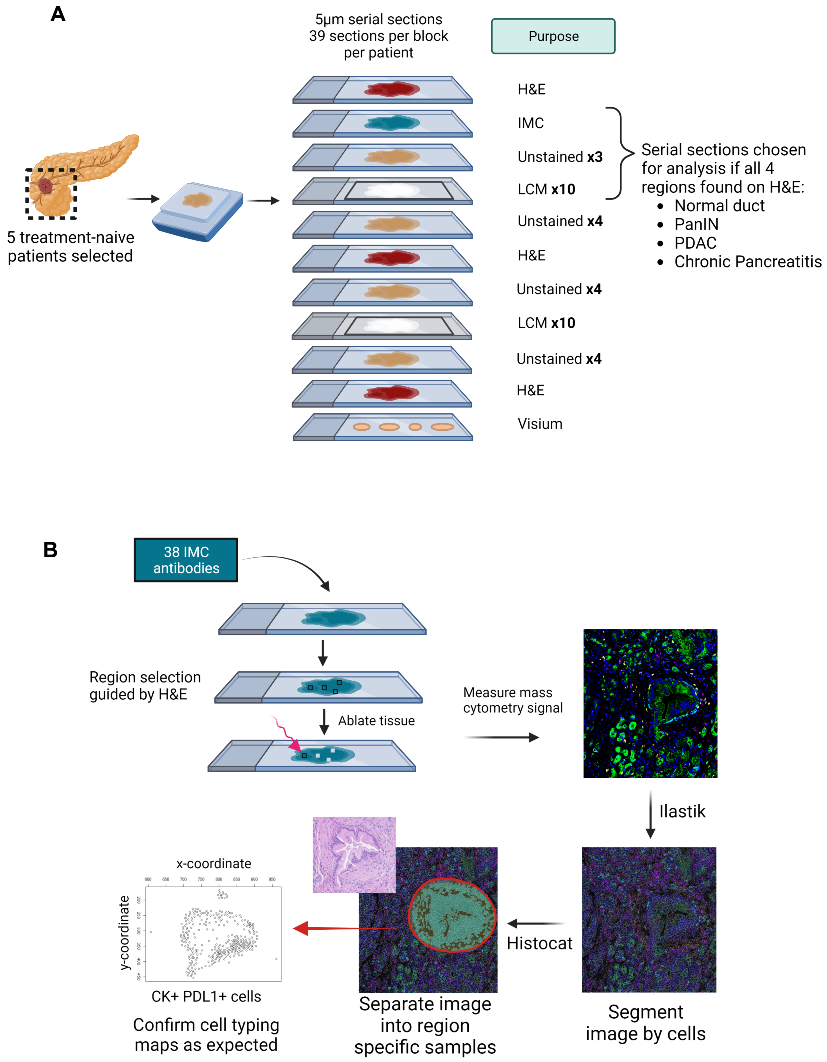

Supplemental Figure 1: A) Serial sections were cut from blocks chosen by an expert pathologist containing 4 regions of interest (normal, PanIN, PDAC and chronic pancreatitis) for analysis using imaging mass cytometry (IMC), immunohistochemistry (IHC), Visium spatial transcriptomics, and laser capture microdissection (LCM) for DNA isolation and subsequent WES analysis. **B)** The step-wise process for staining, segmenting and pre-processing images collected for the imaging mass cytometry analysis. Quality control of segmentation and cell type identities was assured using a matrix of individual cell IDs, their x,y coordinates, and their corresponding phenotypes based on the immune markers (see Supplemental Table 1).

Supplemental Figure 2

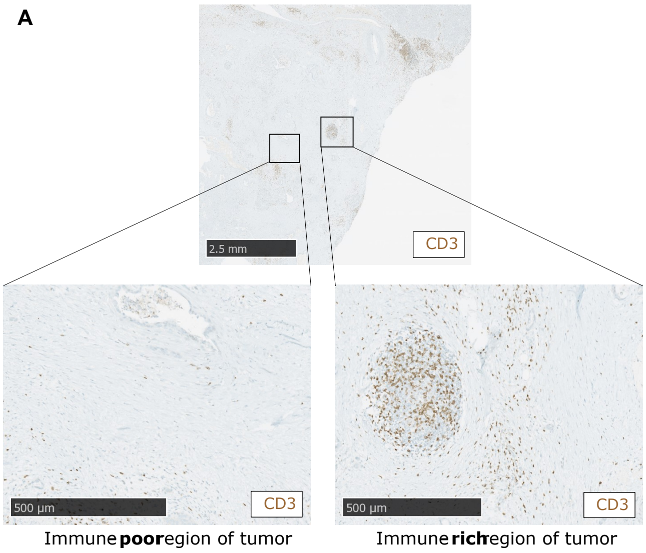

Supplemental Figure 2: Representative IHC staining for CD3^{+} cells in the same tumor section where there are T cell rich regions but also relatively immune-poor regions.

Supplemental Figure 3

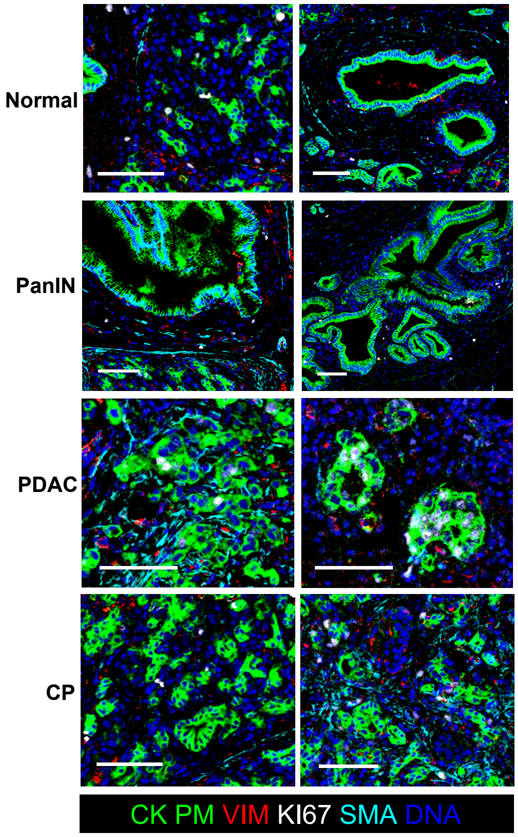

Supplemental Figure 3: Representative IMC images from 2 patients of normal, PanIN, PDAC, and CP regions demonstrating epithelial tissue specific markers (CK – cytokeratin, PM – plasma membrane, VIM – vimentin, KI-67, SMA – smooth muscle actin, DNA). Scale bars: 100μm^2.

Supplemental Figure 4

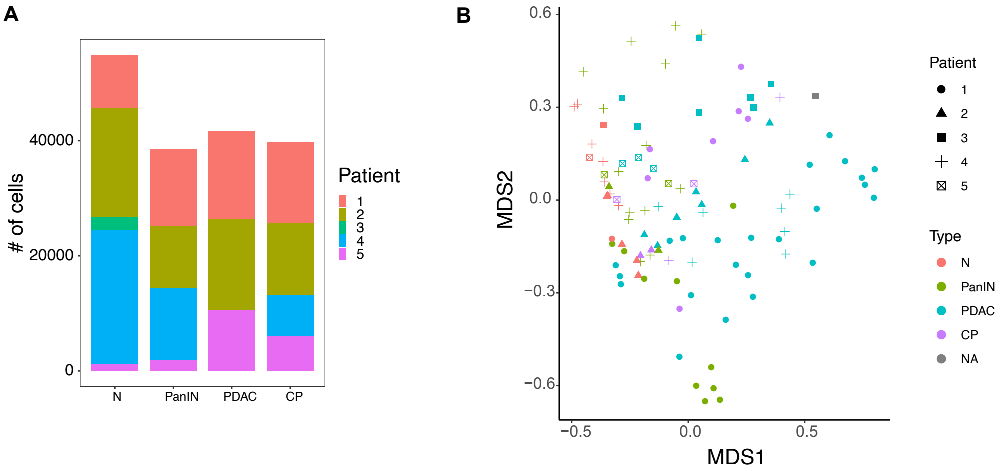

Supplemental Figure 4: A) Contribution of cells from each patient to each region of interest. **B)** Principal component analysis of all IMC samples in the dataset.

Supplemental Figure 5

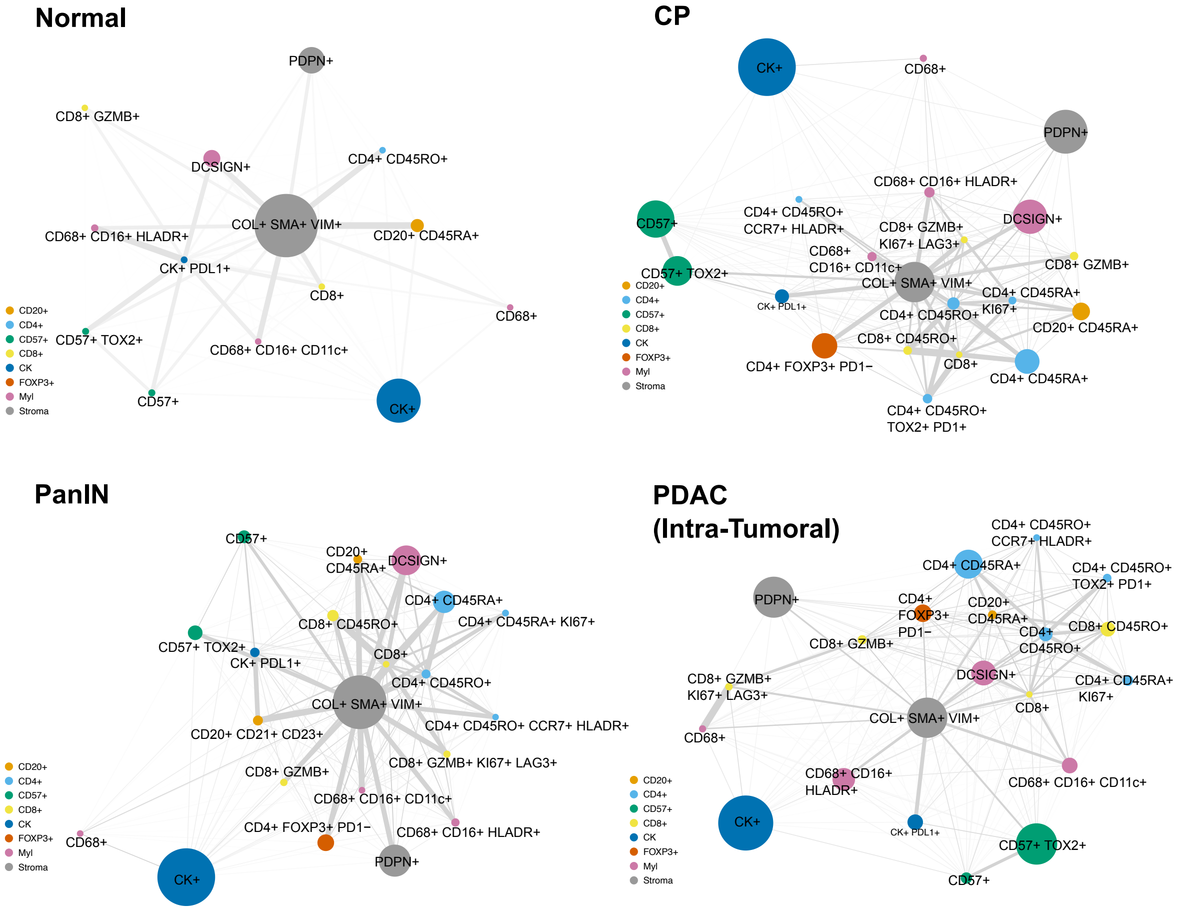

Supplemental Figure 5: Interaction network of normal, CP, PanIN and PDAC regions of all cell types that consisted of at least 1% of the total cells analyzed. Each node indicates a specific cell type colored by their identity. The node size correlates with the relative abundance of the given cell type in the region of interest. The thickness of the edges connecting cell type nodes correlates with proximity.

Supplemental Figure 6

A

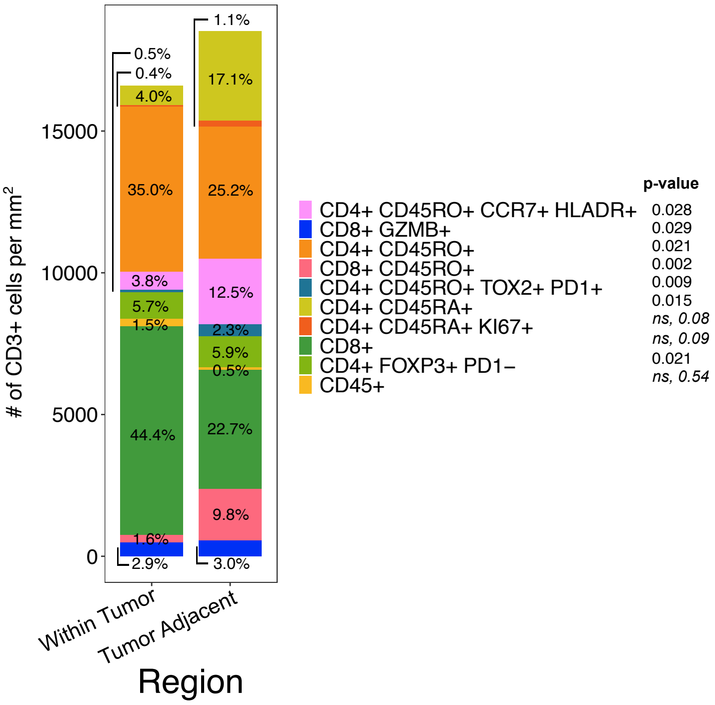

B

Peri-Tumoral

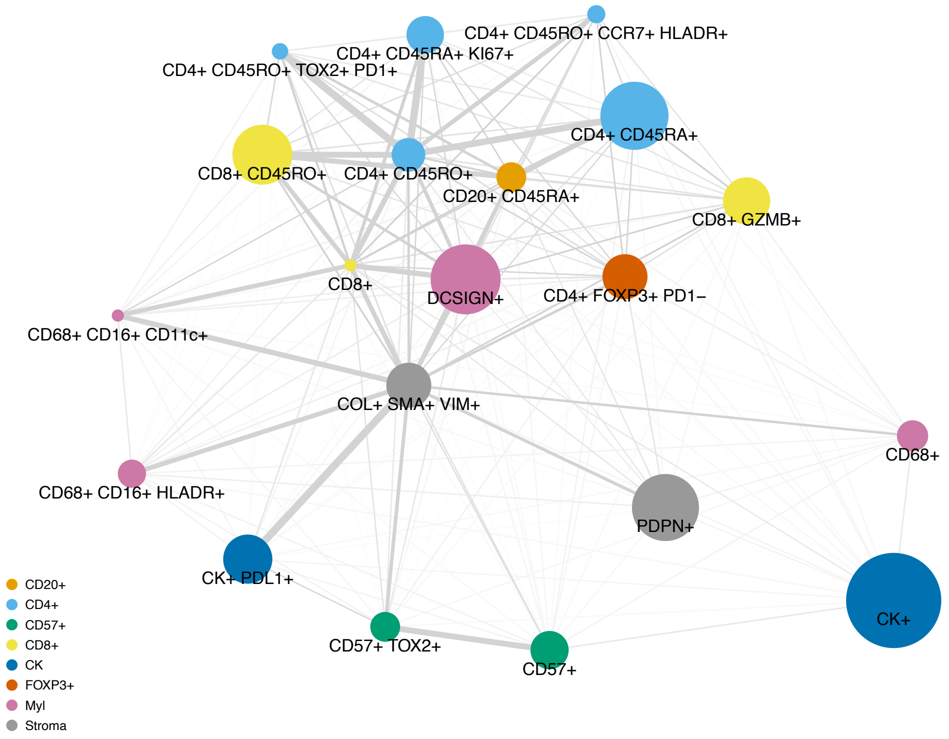

Supplemental Figure 6: A) Quantity of lymphoid cells per mm^2 of tissue analyzed. Each color in the bar indicates the percentage of the total cells that comprise of each specific cell type. Statistics comparing percent composition between within tumor and tumor adjacent regions were determined using a Wilcoxon rank sum test. **B)** Interaction network of the peri-tumoral region. All cell types that consisted of at least 1% of the total cells analyzed. A node indicates a specific cell type colored by their identity. The node size correlates with the relative abundance of the given cell type in the region of interest. The thickness of the edges connecting cell type nodes correlates with proximity.

Supplemental Figure 7

A

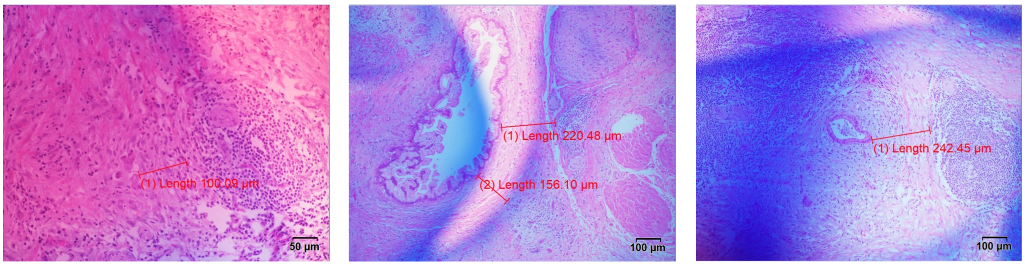

Supplemental Figure 7: Representative images depicting the distance measured by pathologist (E.D.T.) from either PDAC, CP or PanIN regions.

Supplemental Figure 8

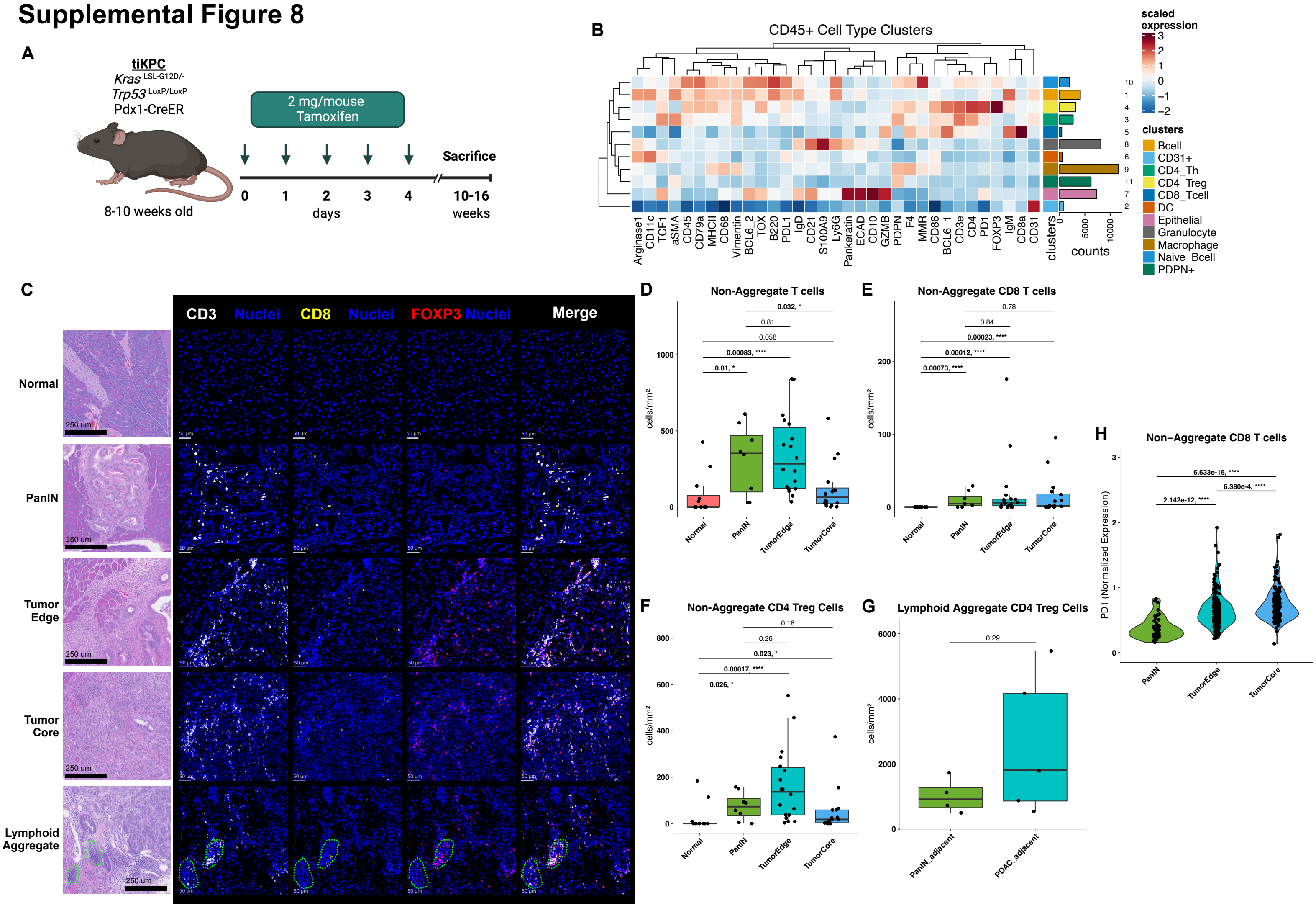

Supplemental Figure 8: Tamoxifen-inducible KPC (tiKPC) mouse model recapitulates spatial features of T cell response to PanIN and PDAC. **A)** Graphical outline of the tiKPC mouse genotype and treatment regimen consisting of daily tamoxifen doses for five days to induce carcinogenesis and sacrifice at 10 to 16 weeks post-induction for tissue collection. **B)** Mean protein expression of immune cell markers among labeled cell types in multiplex immunohistochemical (IMC) profiling of mouse pancreases. Cell types were annotated based on marker gene expression following hierarchical clustering. Lymphoid aggregates are circled in green. **C)** Representative tissue images of T cell markers in normal duct, PanIN, tumor edge, tumor core, and lymphoid aggregates. Hematoxylin and eosin-stained tissues are shown alongside IMC-quantified expression of CD3 (white), CD8 (yellow), FOXP3 (red), and nuclear stain (blue). Lymphoid aggregates are circled in green. **D-F)** Cell type density comparisons across tissue regions in cells/mm2. Densities of all T cells (D), CD4 T regs (E), and CD8 T cells (F) are compared across lesion types. **G)** Density of T regs in lymphoid aggregates proximal to PanIN compared to PDAC. **H)** Violin plots of normalized PD1 protein expression in CD8 T cells across lesion types. Violins are overlayed with points representing expression in individual cell. Densities were calculated using the number cells per mm2 of each ROI and compared by Wilcoxon rank sum test. Normalized protein expression was compared by Wilcoxon rank sum test, and calculated p-values were false discovery rate-adjusted accounting for 33 tested proteins. P-value <= 0.0001, 0.001, 0.01, 0.05, or > 0.05 are denoted in the plots with the respective symbols ****, ***, **, *, or ns.

Supplemental Figure 9

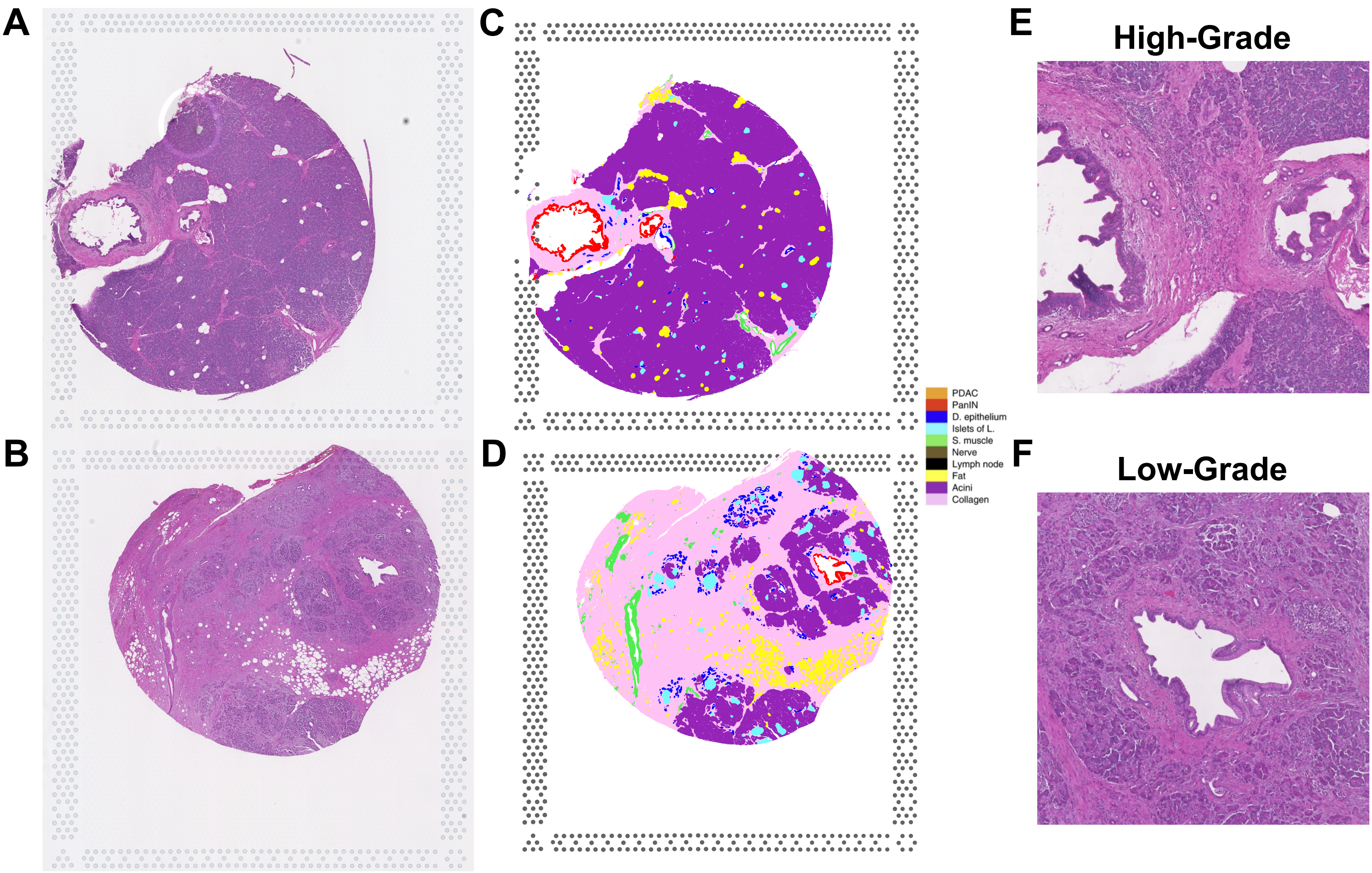

Supplemental Figure 9: Representative images of PanIN lesions in Visium samples. A, B: H&E images of sample PANIN03 containing high-grade PanIN (**A**) and CP05B containing low-grade PanIN (**B**). **C, D**: CODA tissue annotation of PANIN03 (**C**) and CP05B (**D**) images used for spot tissue deconvolution. Colors correspond to PDAC (orange), PanIN (red), ductal epithelium (blue), islets of Langerhans (teal), smooth muscle (green), nerve (brown), lymph node (black), fat (yellow), acini (purple), collagen (pink). **E, F**: Magnified views of representative high-grade (E) and low-grade (F) PanIN lesions.

Supplemental Figure 10

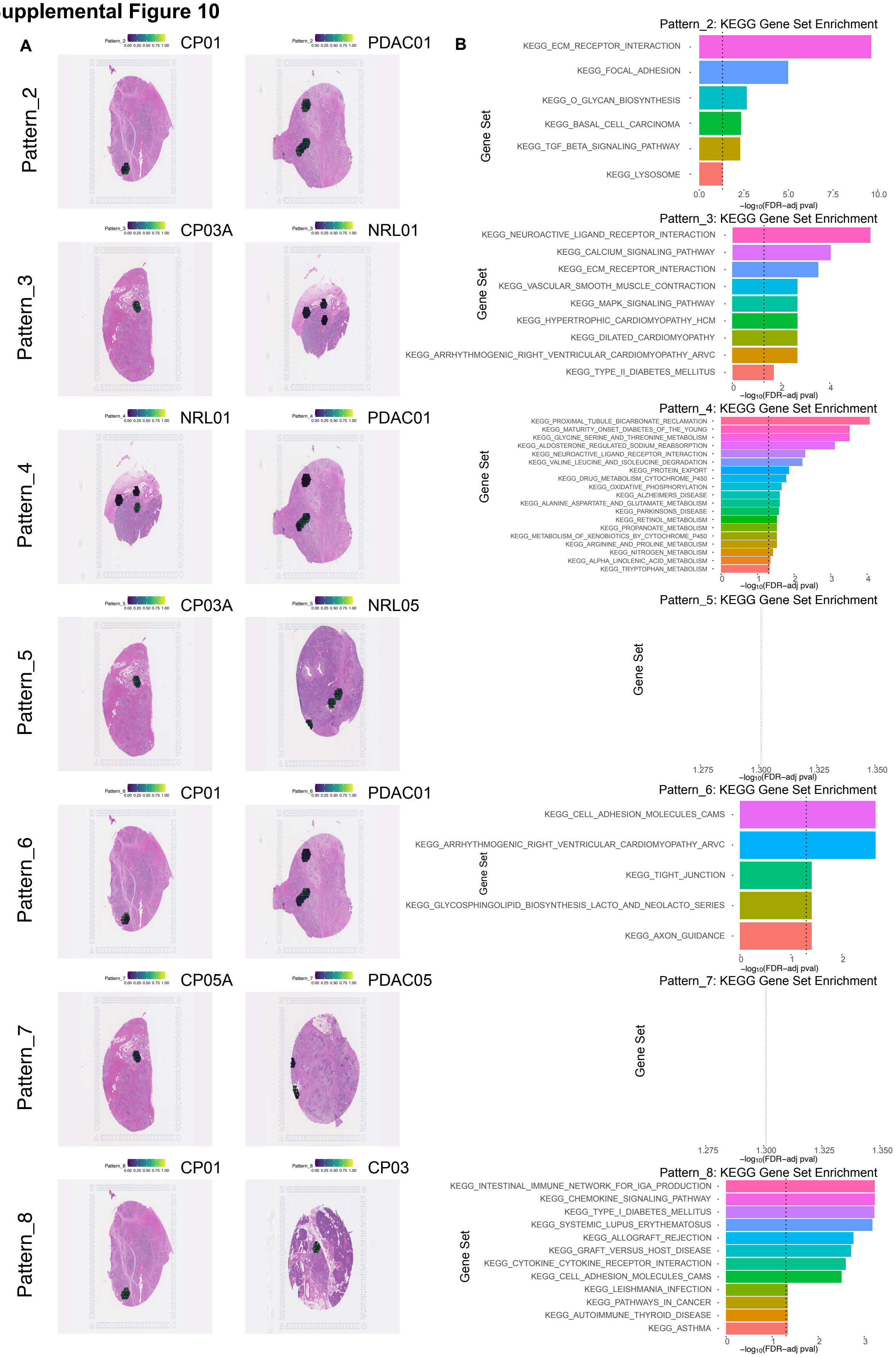

Supplemental Figure 10: Biological features associated with learned CoGAPS patterns. **A)** Representative spatial plots of pattern weights for patterns 2 through 7. **B)** Significantly enriched KEGG gene sets (FDR-adjusted p-value < 0.05) in CoGAPS patterns based on lists of genes ordered by CoGAPS pattern marker score.

Supplemental Figure 11

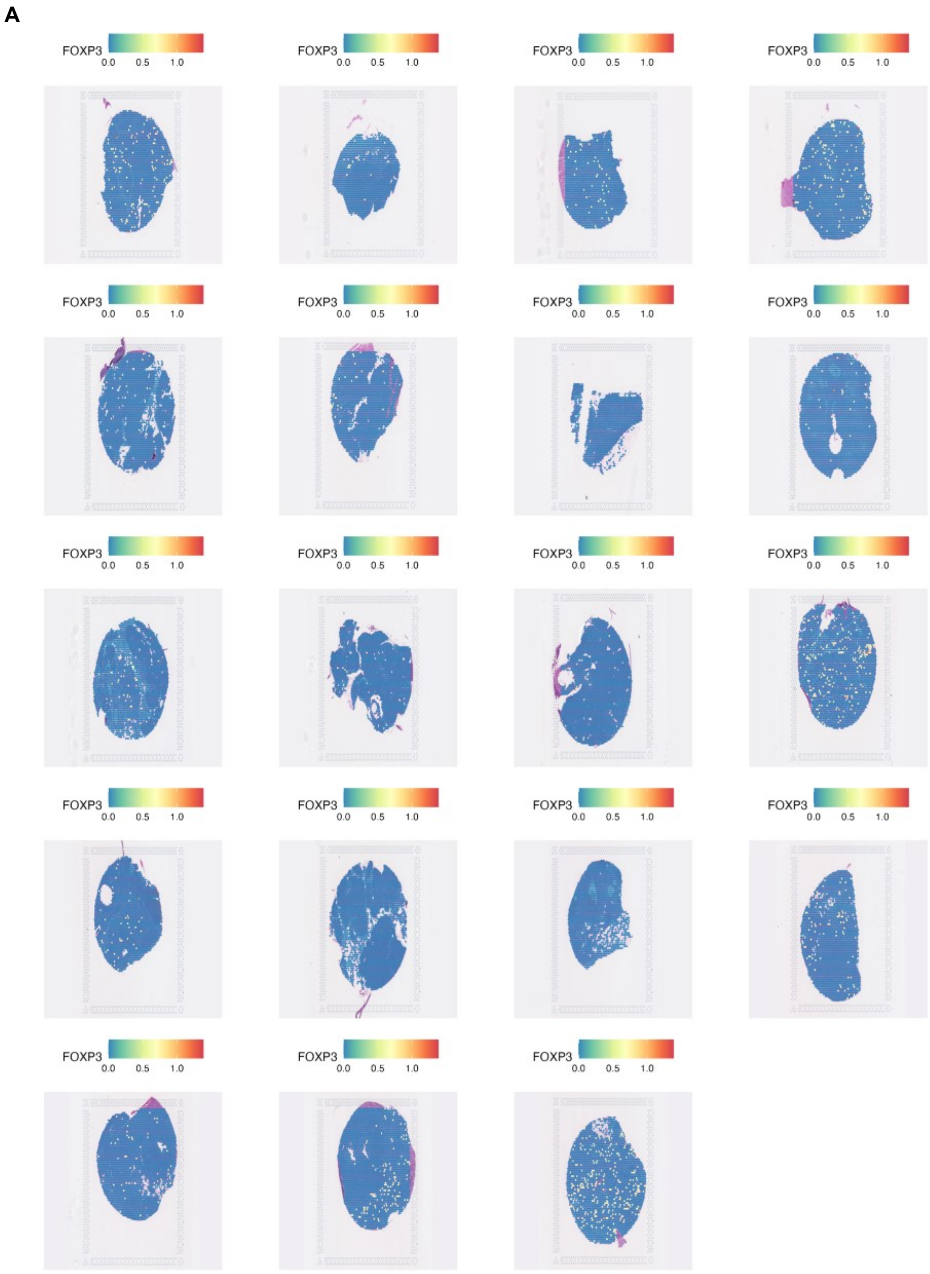

Supplemental Figure 11: Normalized gene expression of FOXP3 across all Visium segments using SC transformation. Sparse expression of this characteristic transcription factor for T regulatory cell identity demonstrates the difficulty of detecting functional T cell states by individual marker gene expression.

Supplemental Figure 12

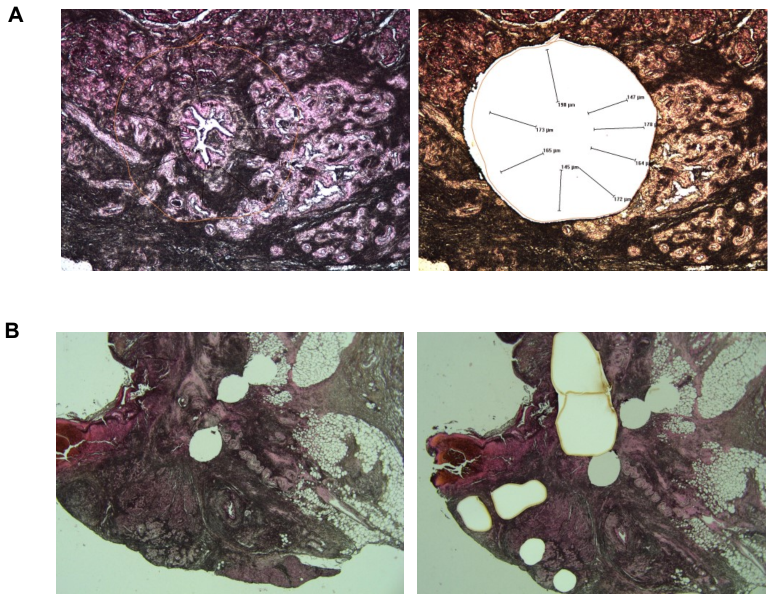

Supplemental Figure 12: A-B) Representative images before and after laser capture microdissection (LCM) of specific regions of PanIN, PDAC, CP and normal tissue for DNA extraction and whole exome sequencing.
